# Tissue-specific reprogramming leads to angiogenic neutrophil specialization and tumor vascularization in colorectal cancer

**DOI:** 10.1172/JCI174545

**Published:** 2024-04-01

**Authors:** Triet M. Bui, Lenore K. Yalom, Edward Ning, Jessica M. Urbanczyk, Xingsheng Ren, Caroline J. Herrnreiter, Jackson A. Disario, Brian Wray, Matthew J. Schipma, Yuri S. Velichko, David P. Sullivan, Kouki Abe, Shannon M. Lauberth, Guang-Yu Yang, Parambir S. Dulai, Stephen B. Hanauer, Ronen Sumagin

**Affiliations:** 1 Department of Pathology, Northwestern University Feinberg School of Medicine, Chicago, Illinois, USA.; 2 Quantitative Data Science Core, Lurie Cancer Center, Northwestern University, Feinberg School of Medicine, Chicago, Illinois, USA.; 3 Department of Radiology, Northwestern University, Feinberg School of Medicine, Chicago, Illinois, USA.; 4 Simpson Querrey Institute for Epigenetics and Department of Biochemistry and Molecular Genetics, Northwestern University, Feinberg School of Medicine, Chicago, Illinois, USA.; 5 Department of Medicine, Gastroenterology and Hepatology, Northwestern Memorial Hospital, Chicago, Illinois, USA.

**Keywords:** Gastroenterology, Immunology, Cellular immune response, Colorectal cancer, Neutrophils

## Abstract

Neutrophil (PMN) tissue accumulation is an established feature of ulcerative colitis (UC) lesions and colorectal cancer (CRC). To assess the PMN phenotypic and functional diversification during the transition from inflammatory ulceration to CRC we analyzed the transcriptomic landscape of blood and tissue PMNs. Transcriptional programs effectively separated PMNs based on their proximity to peripheral blood, inflamed colon, and tumors. In silico pathway overrepresentation analysis, protein-network mapping, gene signature identification, and gene-ontology scoring revealed unique enrichment of angiogenic and vasculature development pathways in tumor-associated neutrophils (TANs). Functional studies utilizing ex vivo cultures, colitis-induced murine CRC, and patient-derived xenograft models demonstrated a critical role for TANs in promoting tumor vascularization. *Spp1* (OPN) and *Mmp14* (MT1-MMP) were identified by unbiased -omics and mechanistic studies to be highly induced in TANs, acting to critically regulate endothelial cell chemotaxis and branching. TCGA data set and clinical specimens confirmed enrichment of *SPP1* and *MMP14* in high-grade CRC but not in patients with UC. Pharmacological inhibition of TAN trafficking or MMP14 activity effectively reduced tumor vascular density, leading to CRC regression. Our findings demonstrate a niche-directed PMN functional specialization and identify TAN contributions to tumor vascularization, delineating what we believe to be a new therapeutic framework for CRC treatment focused on TAN angiogenic properties.

## Abstract 2

Transcriptomic profiling of various tissues and models during the transition from colitis to colorectal cancer reveals contributions of tumor-associated neutrophils to tumor vascularization.

## Graphical abstract

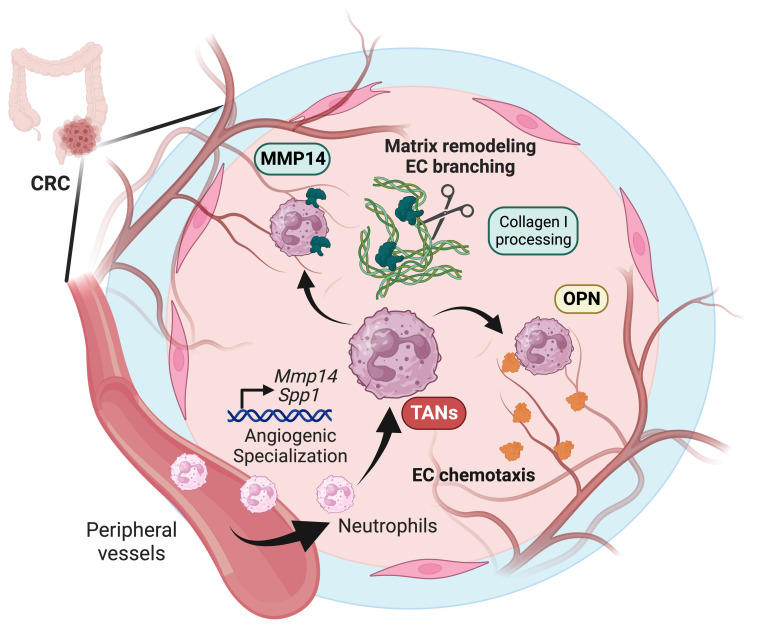

## Introduction

Neutrophils (PMNs) robustly localize to active ulcerative colitis (UC) lesions and the colorectal cancer (CRC) microenvironment. Colitis-associated CRC, in particular, features en masse intratumoral PMN accumulation (1–6). However, across most CRC, PMN burden has been correlated with advanced cancer stage and poor clinical outcomes, including treatment failures and higher risk of distant metastasis (1, 7–9).

The heterogenous nature of circulating and tissue-infiltrating PMNs is now well recognized (10–13), reflecting malleable transcriptional programs of PMNs in inflammation and cancer. The PMN epigenetic landscape also appears not to be silent, as distinct sets of PMN-specific transcriptional factors associated with clusters of transcriptionally active chromatins have been identified (14, 15). As such, tissue PMNs can acquire diverse functional phenotypes, including the reported protumorigenic versus antitumorigenic roles.

We and others have previously reported that PMNs can affect epithelial and tumor cells functions (1, 2). As such, colitis- and CRC-associated PMNs, respectively, promoted tissue damage and antitumorigenic responses during ulceration and early stages of CRC. However, genomic instability caused by persistent PMN presence facilitated tumor adaptation through upregulation of error-prone DNA repair, promoting cancer cell resistance to genotoxic stressors and anticancer therapeutics (1). Surprisingly, although the idea of tissue-specific immune cell polarization resulting in functional diversification is well accepted (16–18), the affect of the CRC niche on tumor associated neutrophils (TANs) remains less explored.

In the current work, we examined how spatial PMN localization to peripheral blood, colitis tissue, and early versus advanced stage cancer niches affected PMN specification, and how this contributed to tumor progression. We identified distinct compartmentalized activation of PMN transcriptomes favoring proangiogenic response in TANs, which, in turn, contributes to vascularization of CRC tumors. An induction of angiogenic programs in PMNs/TANs and their localization to regions of active angiogenesis has been previously seen in transplanted and carcinogenic pancreatic islets (19, 20), melanomas (21), and liver cancer (22). However, most PMN/TAN angiogenic functionalities in these studies were associated with 2 well recognized proangiogenic factors, VEGF and MMP-9. In our study, pathway analysis, targeted screening of differentially expressed genes (DEGs), and functional validation identified the strongest induction of 2 additional angiogenic factors, *Spp1* (Osteopontin/OPN) and *Mmp14* (MT1-MMP), in advanced CRC TANs. Targeting TANs by antibody-mediated depletion, pharmacological inhibition of trafficking, or specific suppression of MMP14 by an allosteric inhibitor potently disrupted tumor vascularization by suppressing new vessel outgrowth, branch architecture, and depth penetration. Such approaches curbed CRC development, identifying promising TAN-centered treatment modalities as an alternative to and/or in additional to traditional antiangiogenic drugs.

## Results

### Tissue compartmentalization of PMN immunophenotypes during the transition from colitis to CRC.

**Figure 1:**
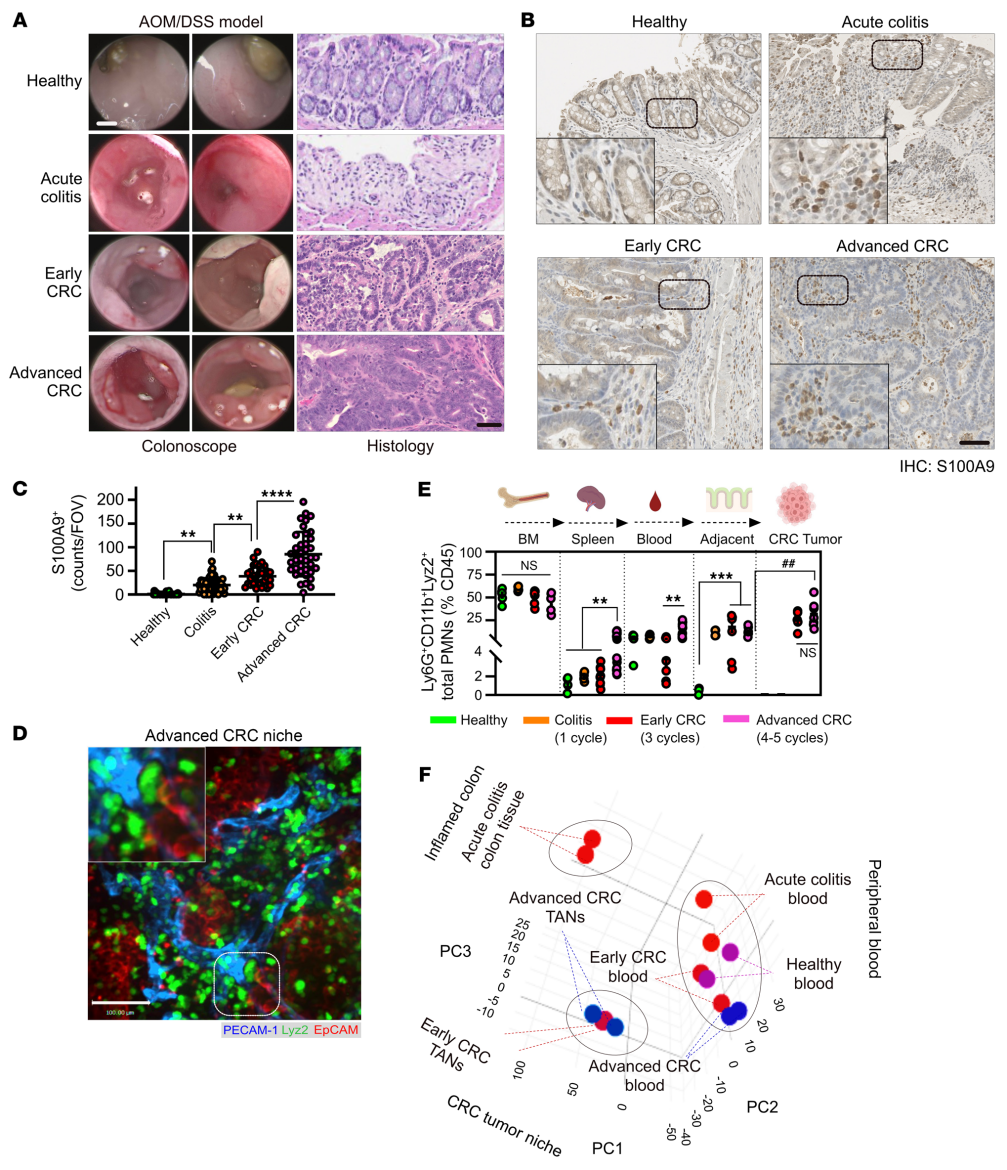
Tissue compartmentalization of PMN immunophenotypes during colitis-to-CRC transition. (**A**) Endoscopic (left) and histological images (right) of healthy, ulcerated and CRC-bearing colons induced by AOM/DSS treatment. Acute colitis 1 DSS cycle, early and advanced CRC with 3 and 5 DSS cycles, respectively. Images representative of *n* = 4 independent experiments. Scale bars: 1 mm for endoscopy and 100 μm for H&E. (**B**) IHC staining and (**C**) quantification of S100A9 PMNs in healthy, inflamed colitis and AOM/DSS induced tumors (*n* = 3–4 mice per condition, with approximately 50 fields of view [FOVs]per condition). Insets are higher magnification images showing PMN accumulation in tumors. Scale bar: 50 μm. (**D**) Whole-mount confocal imaging of AOM/DSS-induced CRC tumors in Lyz2^EGFP^ reporter mice. ECs were visualized by PECAM-1 and tumor cells by EpCAM staining. The inset shows magnified image of area highlighted by dotted line, depicting PMN interacting with blood vessels within the tumor niche. Scale bar: 50 μm. Images representative of *n* = 4 independent experiments. (**E**) Flow cytometric analyses of Ly6G^+^/CD11b^+^/Lyz2^EGFP^ PMN numbers across tissue compartments during CRC development. *n* = 5–8 for healthy and colitis and *n* = 8–12 for CRC. Data are shown as mean ± SEM. **P < 0.01, ***P < 0.001, significance between diseased conditions; ^##^*P* < 0.01, significance between cancer and adjacent tissue(1-way ANOVA with Tukey’s multiple comparison test). (**F**) 3D PCA matrix of RNA-Seq profiles of 7 spatiotemporal conditions. 3 separate PMN clusters (dotted circles) were identified, representing peripheral blood, inflamed colon, and CRC niches.

Given the emerging PMN heterogeneity in inflammation and cancer (10–12, 23), we employed Azoxymethane/Dextran sodium sulfate (AOM/DSS) carcinogen and colitis-driven colon cancer model to spatiotemporally assess the PMN heterogeneity in acutely inflamed colon, low-grade, well-differentiated CRC tumors and advanced high-grade tumors with poorly differentiated histology (24, 25) (Figure 1A and Supplemental Figure 1A; supplemental material available online with this article; https://doi.org/10.1172/JCI174545DS1). This model featured robust and progressively increasing PMN presence during all transitional stages (Figure 1, B and C), culminating in intratumoral, peritumoral, and stromal region TAN accumulation in early and advanced CRC, determined via whole-mount immunofluorescence confocal microscopy imaging on myeloid reporter mice Lyz2^EGFP^ (Figure 1D and Supplemental Figure 1, B and C). Consistently, elevated PMN mobilization to spleen, peripheral blood, and colon tissues or tumors was confirmed by flow cytometry in colitic and tumor-bearing mice (gating on Ly6G^+^/CD11b^+^/Lyz2^EGFP^ PMNs, Figure 1E). The number of Ly6C^hi^/Lyz2^lo^/Ly6G^neg^ monocytes in the peripheral blood of tumor-bearing mice and CD64^+^/Lyz2^int^/Ly6G^neg^ macrophages in CRC tumors remained mostly unchanged (Supplemental Figure 1, D–F).

Phenotypic PMN profiling using established maturity markers revealed the transition from CD101^hi^/CXCR2^+^ mature PMNs in spleen, peripheral blood, and tumor-adjacent tissues to previously reported protumorigenic CD101^lo^/CXCR2^neg^ TANs (Supplemental Figure 1, H and I). Of note, the Ly6G^hi^/CD11b^hi^/Lyz2^hi^ TAN phenotype was distinct from Ly6G^lo^/CD11b^int^/Lyz2^lo^ cells previously referred to as granulocyte myeloid-derived suppressor cells (PMN-MDSCs) (14), which was expanded to a lesser degree in advanced tumors (25%–50% of TANs compared with approximately 10% PMN-MDSCs, Figure 1E and Supplemental Figure 1G).

### Tissue-specific localization drives distinct PMN transcriptional profiles during CRC progression.

Given emerging PMN and TAN phenotypic plasticity, we assessed changes in their transcriptional programs during CRC progression using total-input RNA-Seq profiling of FACS-sorted healthy, colitic and early/advanced CRC-bearing peripheral blood and tissue cells (gating on CD64^neg^/EpCAM^neg^/Ly6G^hi/int^/Lyz2^hi^/Gr1^+^ PMNs/TANs, Supplemental Figure 2A). PMN purity following sorting was confirmed at greater-than 96% by immunofluorescence and following sequencing at transcript level based on established immune gene sets (Supplemental Figure 2, B–D).

A 3D principal component analysis (PCA) separated PMN transcriptomes into 3 distinct groups based on their localization to blood, inflamed colon, and tumors (Figure 1F). Greatest distances in DEG variance were seen in colitis PMNs and TANs relative to peripheral blood PMNs. Meanwhile, peripheral blood PMNs from healthy, colitis, or tumor-bearing mice clustered together with relatively minimal separation within the PCA matrix and correlation dendrograms (Figure 1F and Supplemental Figure 2E). This indicates rapid PMN adaptation upon entering diseased tissue.

**Figure 2:**
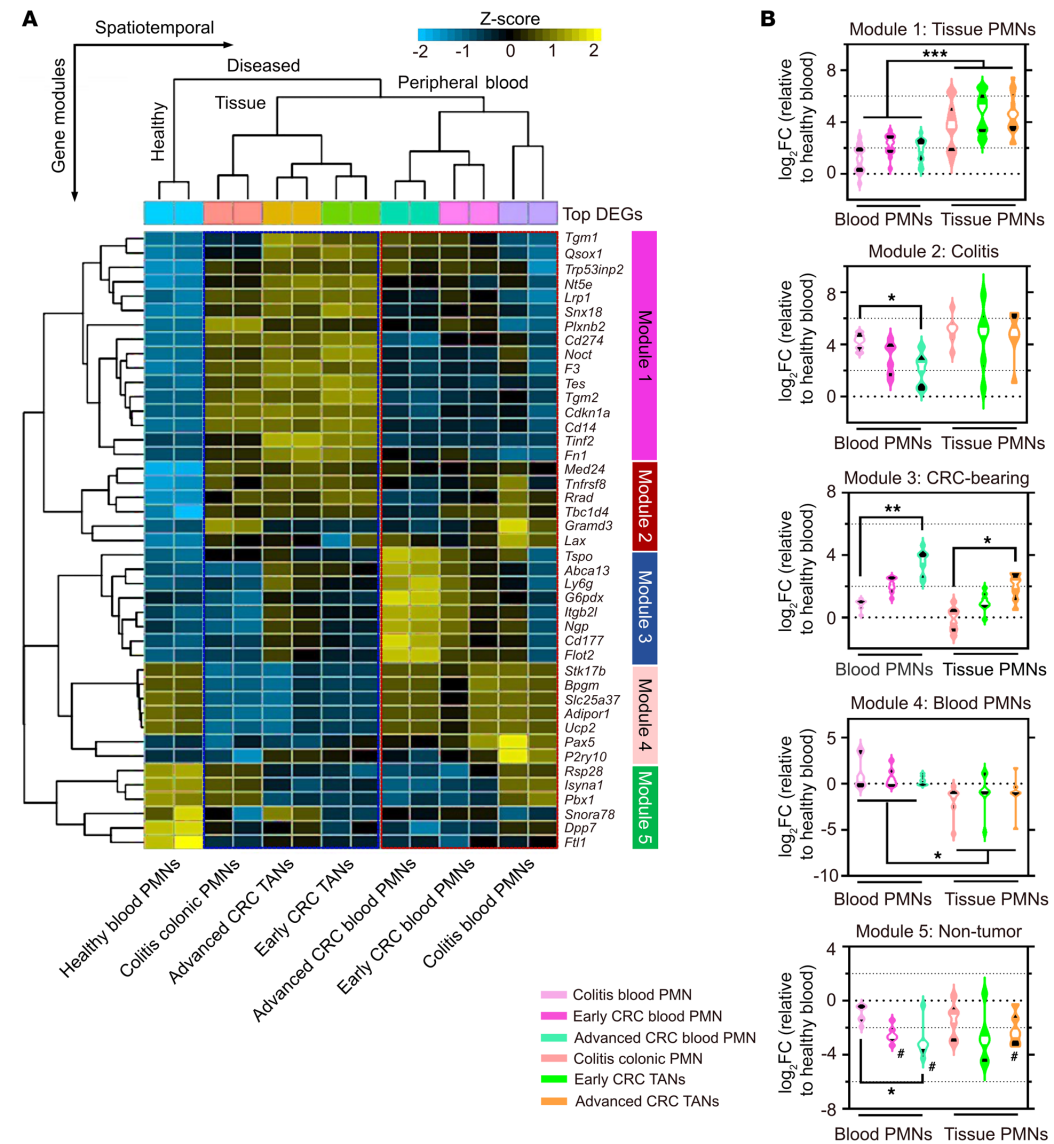
Distinct transcriptomic signatures define PMN/TAN tissue localization. (**A**) Unsupervised hierarchical clustering of the top 50 DEGs with Benjamini-Hochberg’s correction (FDR < 0.05). Row-scaled heatmap representation. (**B**) Violin plots showing expression of DEGs in each module. Middle lines indicate median values (white) with 25th to 75th quartiles (black). *P < 0.05, **P < 0.01, ***P < 0.001, significance between diseased conditions; ^#^*P* < 0.05, decreased compared with healthy blood, 2-sided Mann-Whitney U test.

Unsupervised hierarchical clustering of the top 50 DEGs clearly separated PMN gene signatures based on spatial localization. Five gene modules were identified, including a common gene set separating (a) all peripheral blood (*module 4* in Figure 2A) from (b) colitic and tumor tissue (*module 1*) PMNs/TANs; (c) a distinct colitis signature common to both inflamed blood and tissue condition (*module 2*); (d) a distinct CRC blood PMN signature (*module 3*), and (e) a healthy blood PMN signature (*module 5*) (Figure 2A and Supplemental Figure 2G). Distribution quantification of DEG FC in each module revealed a transcriptional switch as circulating PMNs transitioned to tissue PMNs and TANs as evidenced through downregulated peripheral blood signature in *module 4*, and enrichment of colitic and CRC in *modules 2* and *3* (Figure 2B). Consistently, row-scaled expression analyses with K-means clustering revealed previously established TAN-associated gelatinase/tertiary granule genes (*Mmps12/13, Adam8*) to be enriched in advanced CRC tumors compared with secondary granule genes (*Ltf, Mmps8/25, Adams10/15*) associated with blood PMNs (10, 14) (Supplemental Figure 3A). Using this approach, TANs were clearly distinguished from blood and colon tissue PMNs based on their expression of the established TAN markers *Cd14*, *Cd9*, *Klf6*, and *Runx1* (Supplemental Figure 3C). Circulating PMNs in advanced CRC–bearing mice displayed gene signature previously associated with PMN-MDSCs (14) (*Ltf*, *Npg*, *Camp*, and *Lcn2*), whereas matched TANs displayed more proteolytic (*Adam8*, *Ctsb*, and *Mmp12)* and secretory gene signatures (*Ccl4*, *Cxcl1*, and *Il1b*, Supplemental Figure 3, A and B). Further DEG mapping for PMN/TAN transcription factors (TFs) (10, 14, 15) identified unique signatures representing TAN (*Irf5*, *RelB*, and *Nfkb1/2*), colitic PMN (*Irf6*), and blood PMN (*Cebpd* and *Cebpe*) TFs, as well as distinct surface markers, including CSF3R and CD44 (Supplemental Figure 3, C–E).

### CRC niche drives proangiogenic transcriptional programming in TANs.

**Figure 3:**
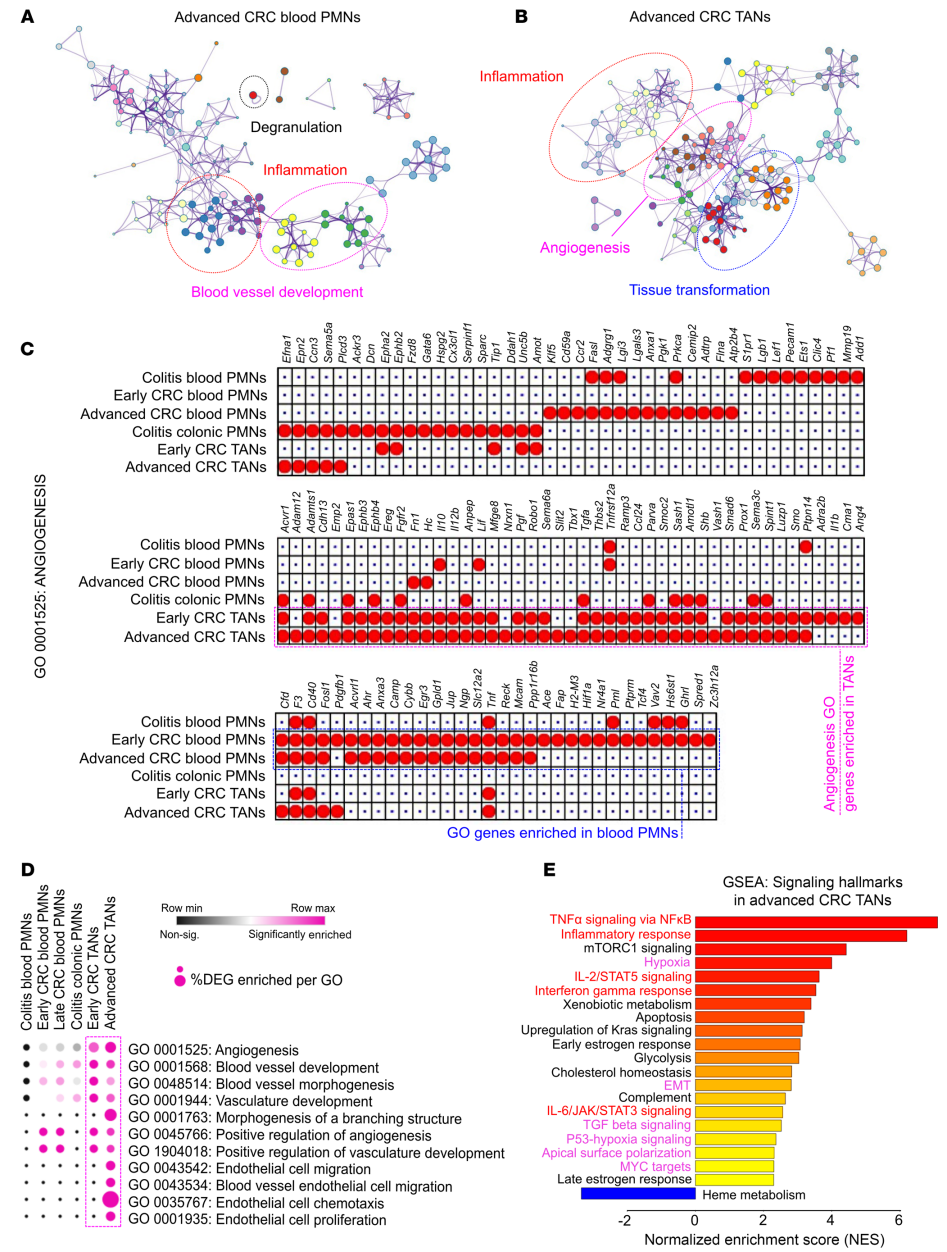
CRC niche drives proangiogenic transcriptional programming in TANs. (**A**) GO network analyses of enriched biological processes in peripheral blood PMNs and (**B**) TANs in advanced CRC. Node size reflects significance of the enrichment test where the edges reflect overlap of GO terms involved in connected biological processes. (**C**) Analysis of GO:0001525 (Angiogenesis). Dotted outlines show enriched DEGs in TANs (magenta) and blood PMNs (blue). (**D**) GO terms enriched for the indicated PMN conditions. Significance of enrichment (FDR < 0.05) was indicated in magenta. The size of each dot shows the percentage of DEGs enriched in each of the specified GO terms. Magenta arrows indicate GO terms associated with vasculature development, angiogenesis, and endothelial cell functions. (**E**) GSEA and overrepresented pathways in advanced CRC TANs. Pathways were ranked based on normalized enrichment score (NES) with adjusted FDR < 0.05 following Benjamini-Hochberg’s correction. Inflammatory and angiogenesis-related pathways are highlighted in red and magenta, respectively.

Next, the differential functions of blood PMNs and CRC TANs were inferred using gene ontology (GO) network analyses. Central gene nodes of blood PMNs from CRC-bearing mice were overrepresented by degranulation, inflammation, and metabolic pathways (Figure 3A and Supplemental Figure 4A). In contrast, inflammation GO gene nodes were reduced in TANs and were superseded by nodes governing tissue transformation and proliferation (Figure 3B and Supplemental Figure 4B). Intriguingly, GO terms of both blood PMNs and TANs were overrepresented by pathways regulating blood vessel morphogenesis and vasculature development. However, TANs were exclusively enriched in angiogenesis GO annotation GO:0001525 (Figure 3B), suggesting potential angiogenic specialization.

Consistently, the number of DEGs associated with the angiogenesis term GO:0001525 was higher in TANs than in blood PMNs (47–52 versus 33–35 genes) or colitic blood and tissue (22 and 31 genes, respectively, Figure 3C). Ancestor chart scoring for vasculature development and angiogenesis revealed enrichment of terms involved in endothelial cell (EC) chemotaxis, morphogenesis of branching structures, and to a lesser extent, EC proliferation in TANs (Figure 3D and Supplemental Figure 4C). In contrast, blood PMNs in healthy, colitic, and CRC-bearing mice showed minimal representation of vessel-regulating functions (Figure 3D). The TAN proangiogenic profile in advanced CRC was further corroborated by Gene Set Enrichment Analyses (GSEA) of the established ‘50 Hallmarks’ gene set (26), revealing positive enrichment for *hypoxia*, *TGF-*β *signaling,* and *Angiogenesis* (Figure 3E). Together, these data suggest that TANs acquire a proangiogenic transcriptomic state and potential vascular functionalities.

### TANs promote CRC tumor vascularization.

**Figure 4:**
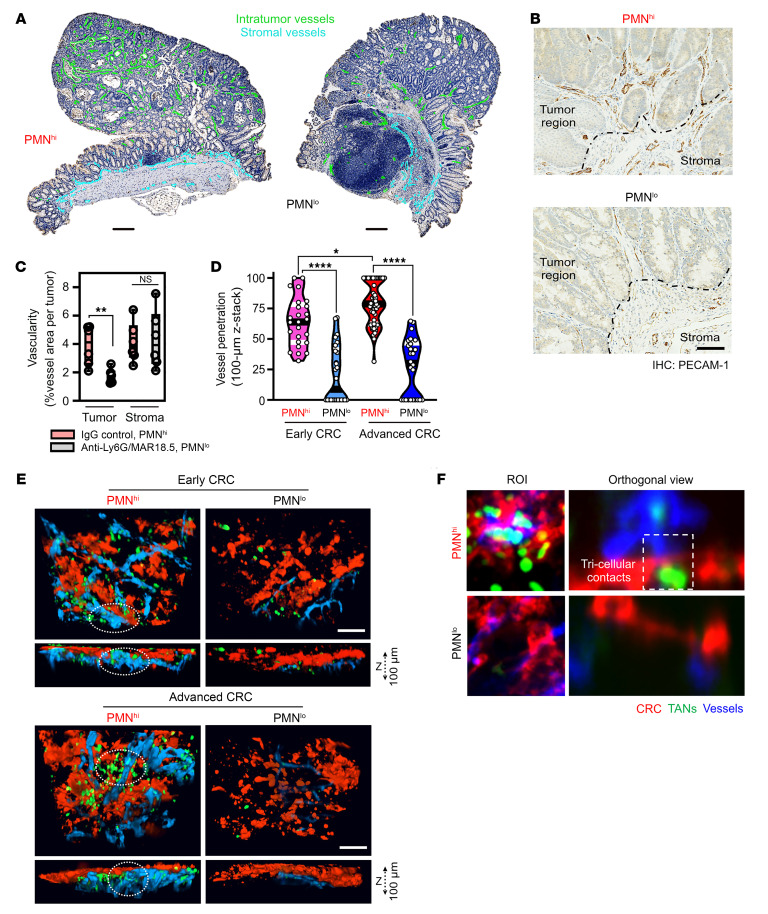
TANs promote tumor vascularization and vessel-tumor depth penetration. (**A**) Representative IHC images of intratumoral (green) and stromal (blue) blood vessels (stained for PECAM-1) in PMN^hi^ (isotype-treated) and PMN^lo^ (treated with anti-Ly6G/MAR18.5) tumors. Scale bar: 200 μm. (**B**) High-power IHC images depict elevated vascular density in PMN^hi^ versus PMN^lo^ tumors. Scale bar: 50 μm. Dashed line demarcates intratumor versus stromal regions. (**C**) Quantification of vessel density from images shown in **A** and **B** (*n* = 5 mice and 8–13 whole-tumor sections per condition). (**D**) Quantification of vessel-tumor depth penetration and TAN presence. (**E**) 3D reconstruction (rendering planes in XZ direction) and Z-stack projections of whole-mount tumor tissue in early and advanced PMN^hi^ versus PMN^lo^ tumors. For each image, 100 μm stacks were generated using 1 μm-focal depth steps. (**F**) Orthogonal views used for vessel depth penetration analyses (right) depicting a tricellular contact between TANs (green), invading blood vessels (blue), and the CRC interface (red) (*n* = 4–6 mice per condition with 50–70 z-stacked images analyzed). Scale bar 10 μm. *P < 0.05, **P < 0.01, ****P < 0.0001, 1-way ANOVA with Tukey’s multiple comparison test.

Given the observed angiogenic transcriptional specialization, we next asked whether TANs promote tumor vascularization. To address this, tumor vessel density was quantified with and without TAN depletion. Depletion protocol using increasing dosing of anti-Ly6G mAb (50–100 μg, daily i.p.) followed by secondary crosslinking anti-rat antibody MAR18.5 (50 μg, every other day i.p.) has been previously described (1) (27). Over 80% TAN depletion was achieved as quantified (Supplemental Figure 5, A and B). TAN depletion (PMN^lo^ tumors) but not isotype control treatment (PMN^hi^ tumors) led to 2-to-3–fold decrease of vessel density in tumors but not in adjacent stromal regions (Figure 4, A–C), confirming TAN contribution to tumor vascularization. The Cancer Genome Atlas (TCGA) database mining of 592 colorectal adenocarcinoma patient cohort (COAD, Pan-Cancer Atlas/cBioPortal) (28) consistently linked high expression of EC PECAM-1 and PMN/TAN S100A8 with poor prognosis and disease outcomes (Supplemental Figure 5C). Further, expression of RNASeq–identified TAN surface markers (*CD86*, *CD14*, *CD74*, and *CD33*), secondary granules (*S100A8* and *S100A9*), and TFs (*SPI1* and *RUNX1*) significantly correlated with 3 major EC markers (*PECAM-1*, *CDH5,* and *VWF*; Supplemental Figure 5, D and E).

We next hypothesize that TANs are required for the spatial organization of tumor vasculature. Thus, ex vivo whole-mount tumor confocal microscopy and 3D reconstruction of confocal images following in situ fluorescence labeling of blood vessels (i.v. administration of anti-PECAM-1 antibody), confirmed a robust vascular disruption and decreased invasion depth of tumor vessels (10–30 versus 70–90 μm, respectively) in PMN^lo^ compared with PMN^hi^ tumors (Figure 4, D and E). Frequent tricellular contacts between TANs, tumor ECs, and cancer cells were detected in PMN^hi^ but, expectedly, not in PMN^lo^ tumors (representative ROI [*left*]and orthogonal view [*right*], Figure 4F). As such, TANs facilitate architectural remodeling and development of CRC tumor vascular network.

**Figure 5:**
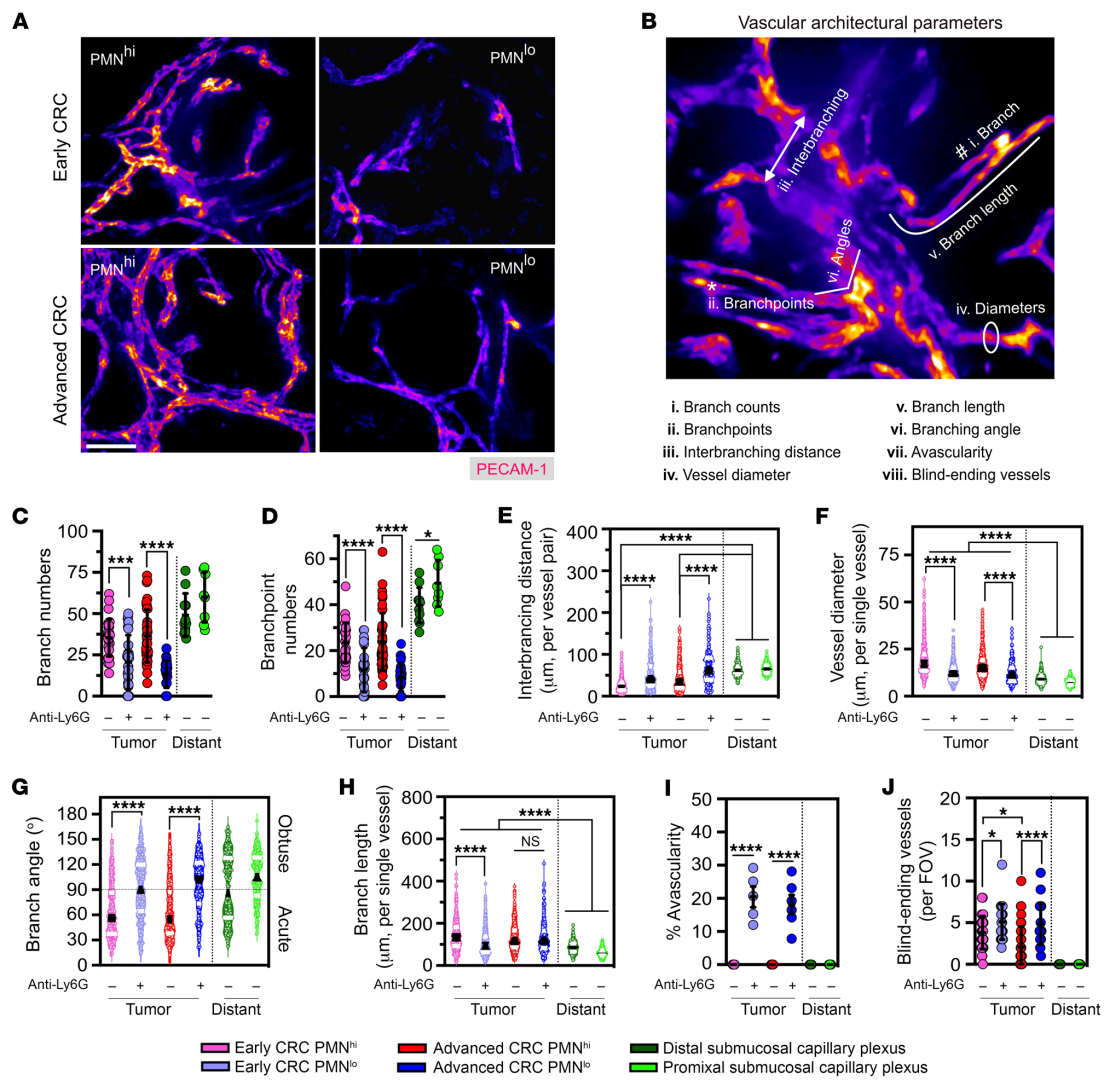
TANs modulate multimodal parameters of vascular architecture and spatial organization. (**A**) Representative confocal microscopy images of whole-mount tumor vasculature (stained for PECAM-1) in early and advanced PMN^hi^ versus PMN^lo^ tumors. Scale bar: 50μm. Images are representative of *n* = 6 tumors with 50–70 FOVs per condition. (**B**) Illustration of 8 architectural parameters assessed for vascular architecture within tumors and tumor-adjacent regions. (**C**–**J**) Quantification of architectural and spatial organization factors constructing the vasculature observed in PMN^hi^ versus PMN^lo^ tumors and the nontumor submucosal capillary plexus. For panels **C**–**H**, each data point represents an individual FOV (600–1,000 vessels analyzed from 50–70 FOVs from 6–8 mice/condition) shown as distribution of the indicated parameters with median values (black line) and 25th to 75th quartiles (white line). For panels **I** and **J**, each data point represents a tumor-bearing mouse with combined FOV analysis from all tumors. *P < 0.05, ***P < 0.001, ****P < 0.0001, 1-way ANOVA with Tukey’s multiple comparison test.

Since tumor vasculature differs from healthy vessels by being more tortuous and disordered (29, 30) (Supplemental Figure 5, F–H), we evaluated the impact of TANs on tumor vascular architecture. Analyses by confocal microscopy of resected tumors showed strong vessel-disrupting effects of TAN depletion in both early and advanced CRC (Figure 5A). We next scored the vascular development parameters (31) as follows: (a) number of branches, (b) branch points, (c) branch lengths, (d) vessel diameters, (e) distance between adjacent branches, (f) branching angles as an index of vessel tortuosity, (g) presence of blind-ending vessels as an index of defective angiogenesis, and (h) avascularity as an index of complete hypoxia (schematic, Figure 5B and Supplemental Figure 5F). Both early and advanced CRC tumor vessels displayed highly irregular and sharp-angled branches with variable lengths compared with more homogeneously structured branches in healthy colon mucosa (Supplemental Figure 5G). Vessel diameters, branch numbers, and branchpoints were markedly reduced in PMN^lo^ tumors, whereas interbranching distances and branching angles were substantially elevated, indicating reduced complexity of branching structures and tortuosity leading to vessel relaxation (Figure 5, C–G). Branch lengths was reduced with TAN depletion only in early tumors (Figure 5H). The number of blind-ending vessels and avascular regions, frequently associated with tissue hypoxia and necrotic cell death, were significantly increased in PMN^lo^ tumors (Figure 5, I and J).

### Angiogenic TANs express high levels of MMP14 and SPP1/OPN.

**Figure 6:**
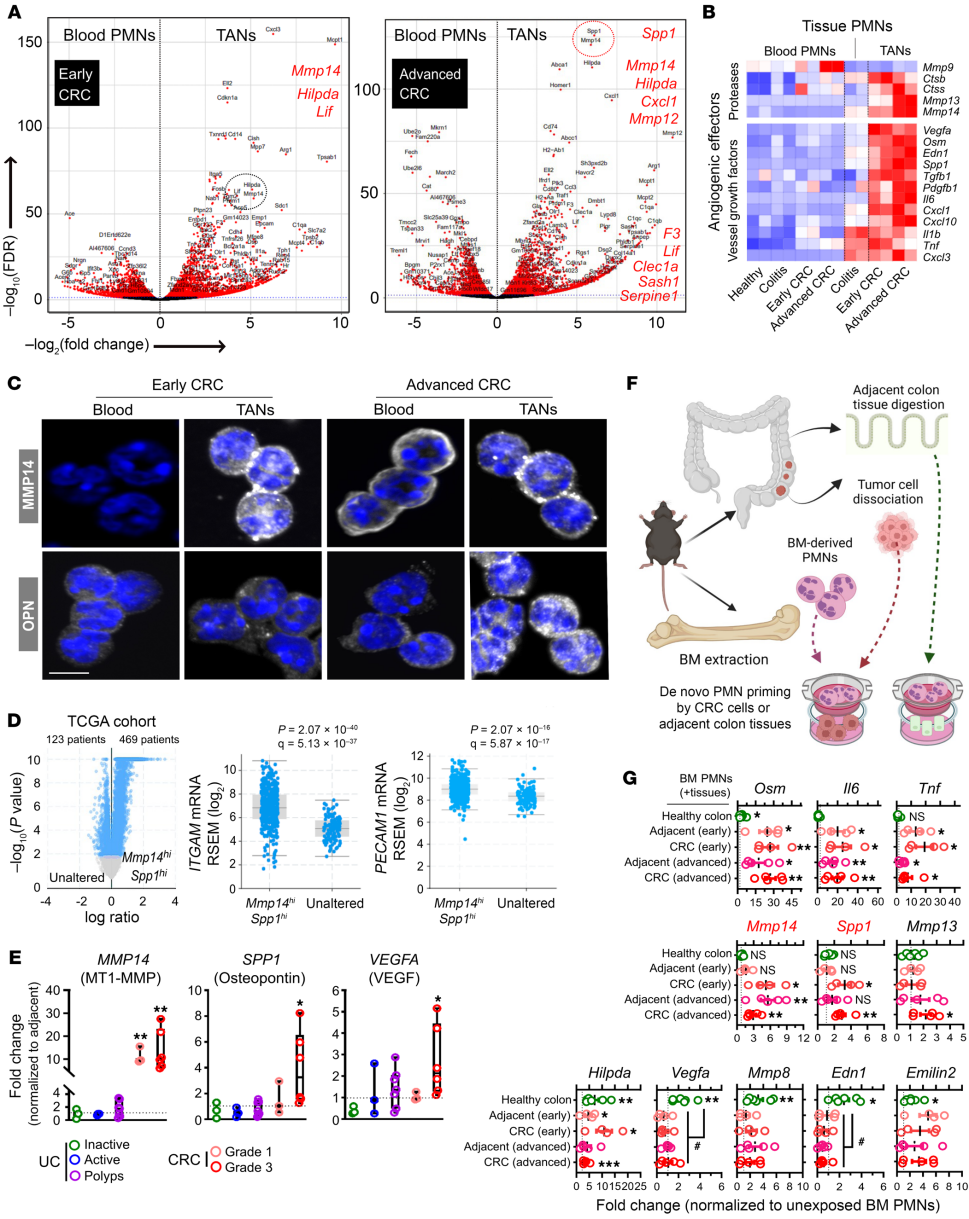
Angiogenic TANs express high levels of MMP14 and SPP1/OPN. (**A**) Volcano plots of DEGs (red) enriched in peripheral blood PMNs and CRC TANs from early (left) and advanced (right) CRC. Red dotted circle highlights *Spp1* (encoding OPN) and *Mmp14* (encoding MT1-MMP) as top enriched genes in advanced CRC (*P* < 10^–100^). (**B**) Row-scaled heatmap representation of elevated levels of established angiogenic factors including proteases and vessel growth factors in TANs. (**C**) Representative confocal microscopy images of FACS-sorted blood PMNs and TANs from advanced CRC stained for intracellular levels of MMP14 and OPN proteins (white). Nuclei were by Hoechst staining (blue). Scale bar: 5 μm. Images representative of *n* = 4 independent experiments. (**D**) Volcano plot (*left*) stratified COAD patients from Pan-Cancer Atlas (*n* = 592) into altered (*MMP14^hi^*/*SPP1^hi^*, *n* = 469 with log ratio > 1.5, adjusted *P* cutoff = 0.01) or unaltered (*n* = 123 remaining patients) groups. Expression levels of PMN markers *ITGAM*/CD11b (middle) and EC marker *PECAM1* (right) were compared between *MMP14^hi^*/*SPP1^hi^* and unaltered groups by 2-sided student’s *t* test (*P* value and FDR-adjusted *q* value are shown). (**E**) mRNA expression (normalized to adjacent noninflamed or noncancerous tissues) analysis of clinical specimens from UC patients (*n* = 3 inactive UC, *n* = 3 active UC, *n* = 10 severe UC polyps) and CRC patients (*n* = 3 grade 1, *n* = 6 grade 3). (**F**) Schematic and (**G**) mRNA expression analyses of established angiogenic genes in BM-derived PMNs following 24 hours incubation with dissociated healthy colon, CRC, or cancer-adjacent tissue. Gene expression was normalized to unexposed PMNs cultured in the same setup (*n* = 4 independent experiments). *P < 0.05, **P < 0.01, ***P < 0.001 (2-sided student’s *t* test, exposed versus unexposed BM-derived PMNs). ^#^*P* < 0.05, 1-way ANOVA with Tukey’s multiple comparison test.

To identify specific molecular targets that can mitigate the observed TAN impact on tumor vascular development, we performed a pairwise comparison of transcriptional profiles of blood PMNs and TANs. Volcano plot analyses comparing DEGs revealed *Mmp14* (encoding MT1-MMP) and *Spp1* (encoding Osteopontin/OPN) as top candidate genes enriched in advanced CRC TANs (Figure 6A). Of interest, both genes have been previously implicated in angiogenesis and malignant transformation (32–35). Several other angiogenic genes (*F3*, *Serpine1*, and *Mmp12*) were similarly upregulated in TANs (Supplemental Figure 4D), supporting an angiogenic transcriptional state of TANs in advanced CRC. Targeted DEG screening of the angiogenesis/vessel development GO pathways identified additional genes encoding several proteases, vascular growth factors (Figure 6B), and hypoxia-responsive mediators (Supplemental Figure 4D) with well-documented proangiogenic implications (36, 37).

Imaging and flow cytometry analyses (gating strategy shown in Supplemental Figure 6, A–D) revealed overall MMP14 expression to be highly induced in TANs compared with circulating PMNs, with MMP14^hi^ TAN subset detected in both early and advanced tumors, as well as in tumor-adjacent regions (Figure 6C and Supplemental Figure 6, E–G). OPN expression was more restricted to advanced CRC TANs (Figure 6C and Supplemental Figure 6, B and H). Peripheral circulating, tumor-adjacent PMNs and TANs expressed similarly low levels of proangiogenic VEGF and IL-17A in early and advanced disease (Supplemental Figure 6, C, D, I, and J). Consistent with previous reports, MMP14 and OPN expression was also detected on tumor macrophages and EpCAM^+^ tumor cells (38–41), however, at 2- to 3-fold lower levels compared with TANs (Supplemental Figure 6, K, L, O, and P), indicating TANs as a major source of MMP14 and OPN in the CRC tumor niche. Macrophages and tumor cells expressed much high levels of VEGF compared with TANs, while levels of IL-17A were not higher (Supplemental Figure 6, M, N, Q, and R).

To test the clinical relevance of our findings, the COAD cohort of 592 patients (cBioPortal) (28) was stratified based on expression of *MMP14^hi^*/*SPP1^hi^* oncoprint. The majority (*n* = 469) of patients were identified as *MMP14^hi^*/*SPP1^hi^* (altered) and as normal/unaltered (*n* = 123) *MMP14*/*SPP1* expression relative to healthy tissue (Figure 6D). The *MMP14^hi^*/*SPP1^hi^* cohort was associated with the aggressive mucinous adenocarcinoma subtype and advanced or metastatic disease stages (Stage IIIB-C and Stage IVA-B, respectively, Supplemental Figure 6S). Importantly, patients with *MMP14^hi^*/*SPP1^hi^* oncoprint exhibited higher expression of myeloid and TAN (*ITGAM*, *CD14*, *S100A8*, and *S100A9*) or EC (*PECAM1*, *VWF*, and *NOTCH3*) markers (Figure 6D and Supplemental Figure 7, A–C). Complementarily, reverse-transcription quantitative PCR (RT-qPCR) analyses on UC and CRC tissues similarly revealed robust induction of *MMP14*, *SPP1,* and *VEGFA* (Figure 6E), but not of the angiogenic receptors *VEGFR1* and *VEGFR2,* or *TGF1B* (Supplemental Figure 7D) in grade 3 CRC. Thus, analyses of public patient databases and clinical specimens support our animal model findings, effectively linking *MMP14*/*SPP1* expression and TAN presence with tumor vasculature in human cancer.

### Ex vivo simulation of the tumor niche activates the proangiogenic TAN signature in bone marrow-derived PMNs.

We next tested whether the CRC microenvironment promotes proangiogenic TAN specialization. To simulate PMN-to-TAN transition during tumor development, isolated bone marrow–derived (BM-derived) PMNs from early or advanced CRC tumor-bearing mice were exposed to matched early or advanced tumor or tumor-adjacent epithelial cells (experimental setup shown in Figure 6F). Exposure to either tumor or tumor-adjacent cells, but not to healthy colon epithelium, induced PMN activation and significantly upregulated genes encoding proinflammatory cytokines (*Osm*, *Il6*, and *Tnf)* (Figure 6G). Importantly, BM-PMN exposure to early/advanced CRC cells induced a robust *Mmp14* and *Spp1* transcription (Figure 6G). Healthy colon tissue exposure was sufficient to induce activation of several hypoxic (*Hilpda*) and angiogenic genes (*Vegfa,* *Mmp8*, *Osm*, *Edn1,* and *Emilin2*) in PMNs, but not *Mmp14* or *Spp1*. This demonstrates specific transcriptional activation of both genes in PMNs by the CRC niche.

### TAN-derived OPN and MMP14 respectively enhance EC migration and vascular branching.

**Figure 7:**
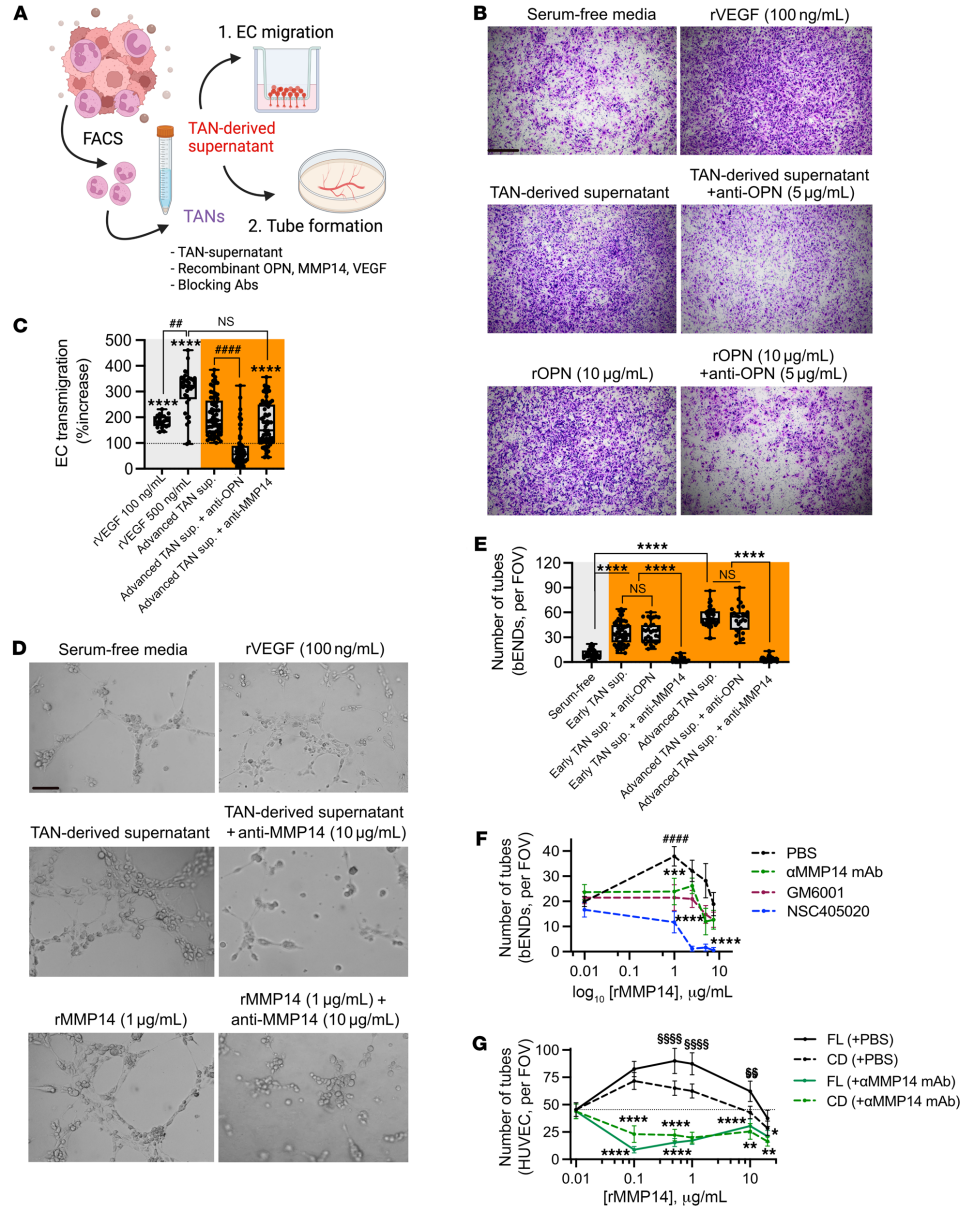
TAN-derived OPN and MMP14 promote endothelial cell migration and vascular branching. (**A**) Schematic of in vitro EC functional assays. (**B**) Representative images of the EC migration assay using cultured bEND cells with or without TAN-derived supernatant (1:1 PMN/EC ratio) or rOPN, rVEGF treatment with or without specific antibody inhibition. Scale bar: 75 μm. (**C**) Quantification of EC migration (relative to serum-free condition) following coincubation with TAN-conditioned media, with or without antibody-mediated inhibition of OPN (6 μg/mL) and MMP14 (10 μg/mL). (**D**) Representative images of EC tube formation assay using cultured bEND cells with or without TAN-derived supernatant treatment (1:3 PMN/EC ratio), or rMMP14 with the indicated antibody inhibition. Scale bar: 10μm. (**E**) Quantification of formed tube numbers per FOVs upon coincubation with TAN-conditioned media, with or without antibody-mediated inhibition of OPN (6 μg/mL) or MMP14 (10 μg/mL). (**F**) Dose-response curves of cultured murine bENDs treated with rMMP14 with or without MMP14 inhibition. (**G**) Dose-response curves of cultured HUVECs following stimulation with catalytic domain (CD) or full-length (FL) rMMP14 (0.01–20 μg/mL), with or without Ab-inhibition of MMP14 (10 μg/mL). For tube formation and EC migration, images are representative of *n* = 2 independent repeats with TAN supernatants isolated from 3 mice for each performed in duplicates. For recombinant protein, *n* = 3 independent repeats performed in triplicates. For all quantifications, 15–20 FOVs were analyzed for each condition. For EC transmigration assay, 2-sided student’s *t* test was performed to compare treatments with serum-free condition. ****P < 0.0001, ^####^*P* < 0.0001, MMP14 blockade versus nonblocking conditions. For tube formation assays in bEND or HUVEC cells, 1-way ANOVA was performed comparing Ab-blockade versus control conditions and between different concentrations of recombinant proteins. *P < 0.05, **P < 0.01, ***P < 0.001, ****P < 0.0001, between treatment conditions. ^§§^*P* < 0.01, ^§§§§^*P* < 0.0001, selected dose(s) relative to the minimal rMMP14 dose of 0.01 μg/mL.

Transcriptomic analyses indicated potential TAN regulation of the tumor vasculature development by enhancing EC migration and de novo vascular branching. To test this, we examined whether CRC TAN supernatants (isolated from advanced tumors) induced EC chemotaxis and tube formation (Figure 7A). Slow-migrating murine ECs (bEND.3) seeded on fibronectin-coated permeable supports were found to efficiently migrate toward CRC TAN supernatants introduced to the bottom chambers. Interestingly, Antibody-mediated OPN but not MMP14 blockade significantly suppressed EC chemotaxis, with recombinant VEGF used as positive control (Figure 7, B and C). Supporting the role of OPN in promoting EC chemotaxis, at several concentrations tested, recombinant OPN (rOPN) induced migration of murine (bEnd.3) and human umbilical vein ECs (HUVEC) (Supplemental Figure 7, E and F). These experiments established that TAN-derived OPN but not MMP14 elicits chemotactic effects guiding EC migration.

We next examined the role of TANs and specific OPN versus MMP14 contributions to de novo vascular branching using tube formation assay. Advanced CRC TAN-conditioned media accelerated de novo assembly of bEnd.3 ECs into highly branched tubal networks, whereas antibody-mediated inhibition of MMP14 but not OPN abolished EC branching and inhibited formation of sprouting vessels (Figure 7, D and E). Consistently, increased concentrations of recombinant MMP14 (rMMP14) but not rOPN effectively enhanced bEND.3 and HUVEC tube formation (Supplemental Figure 7, G and H), establishing a unique role of MMP14 in de novo vessel branching. Supporting clinical significance of our observations, well-established catalytic and allosteric inhibitors of MMP14 activity (pan-MMP inhibitor, GM6001 (42, 43), and NSC405020 (44, 45) effectively curbed rMMP14-induced vessel network formation in murine ECs (Figure 7F and Supplemental Figure 7G). The inhibitory impact of NSC405020 was more pronounced compared with GM6001 at all tested concentrations. Consistently, antibody-mediated inhibition of MMP14 significantly reduced EC branching induced by the full-length (FL) or the catalytic domain (CD) of rMMP14 in HUVECs, with FL-rMMP14 driving more robust tube formation (Figure 7G and Supplemental Figure 7H). Of note, high concentrations of rMMP14 (5.0–7.5 μg/mL in bEND.3 and 10–15 μg/mL in HUVEC) produced lesser effect on EC branching compared with lower concentrations (approximately 1.0 μg/mL for both bEND.3 and HUVEC, Figures 7, F and G), indicating a narrow range for optimal activity of rMMP14. Collectively, these findings demonstrate 2 nonoverlapping angiogenic activities of TANs, with OPN promoting EC chemotaxis and MMP14 accelerating vascular network formation.

### Targeting PMN recruitment and TAN angiogenic actions to disrupt tumor vasculature and induce CRC regression.

**Figure 8:**
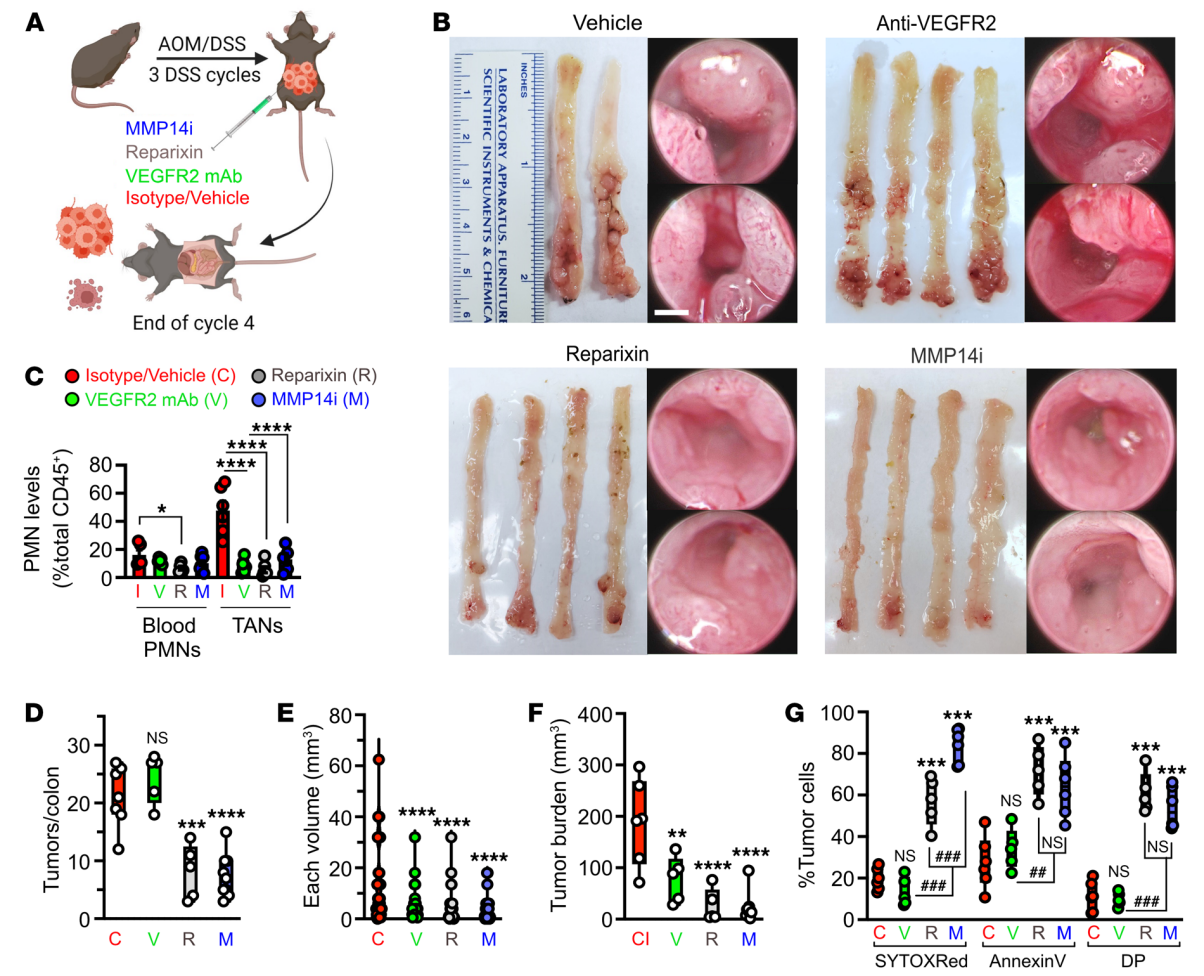
Pharmacological inhibition of TAN recruitment or MMP14 inhibition promotes CRC regression. (**A**) Schematic of the treatment regimens. (**B)** Representative macroscopic images of excised colons and high-resolution endoscopic images of advanced CRC (week 15) treated with either vehicle, anti-VEGFR2 neutralizing mAb (clone DC101(49), 50 mg/kg/day, 3 times/week), CXCR2 inhibitor, Reparixin (5 mg/kg/day (46, 47), daily) or MMP14 allosteric inhibitor NSC 405020 (2.0 mg/kg/day (45), 3 times/week). Scale bar: 1mm. (**C**) Flow cytometry analyses of Ly6G^+^/CD11b^+^/Lyz2^hi^ blood PMN and TAN numbers following indicated treatments (*n* = 6–9 mice/treatment). (**D**) Quantifications of tumor penetrance, (**E**) individual tumor volume, (**F**) tumor burden (cumulative tumor volume), and (**G**) cell death indicated by % Annexin V^+^ and SYTOX-Red^+^ following flow cytometry analysis (Vehicle/ isotype, *n* = 9; Anti-VEGFR2, *n* = 6; Reparixin, *n* = 6; MMP14, *n* = 9 mice; 1-way ANOVA with Dunnett’s multiple comparison test). *P < 0.05, **P < 0.01, ***P < 0.001, ****P < 0.0001, 1-way ANOVA with Tukey’s multiple comparison test.

To test whether inhibiting PMN tumor infiltration or TAN proangiogenic activity can be used as therapy to curb tumor growth, we utilized commercially available small molecule inhibitors Reparixin (a CXCR2 inhibitor) (46–48) and NSC405020 (allosteric MMP14 inhibitor) (45). Anti-VEGFR2 neutralizing antibody (clone DC101) with established antitumorigenic activity (49) was used as a reference control. All 3 treatment regimens, simulating antitumor therapy, were initiated at the start of DSS cycle 4 (once tumors are established and progressed) and maintained through the endpoint (schematic, representative tumor images and histology shown in Figure 8, A and B and Supplemental Figure 8, A and B).

**Figure 9:**
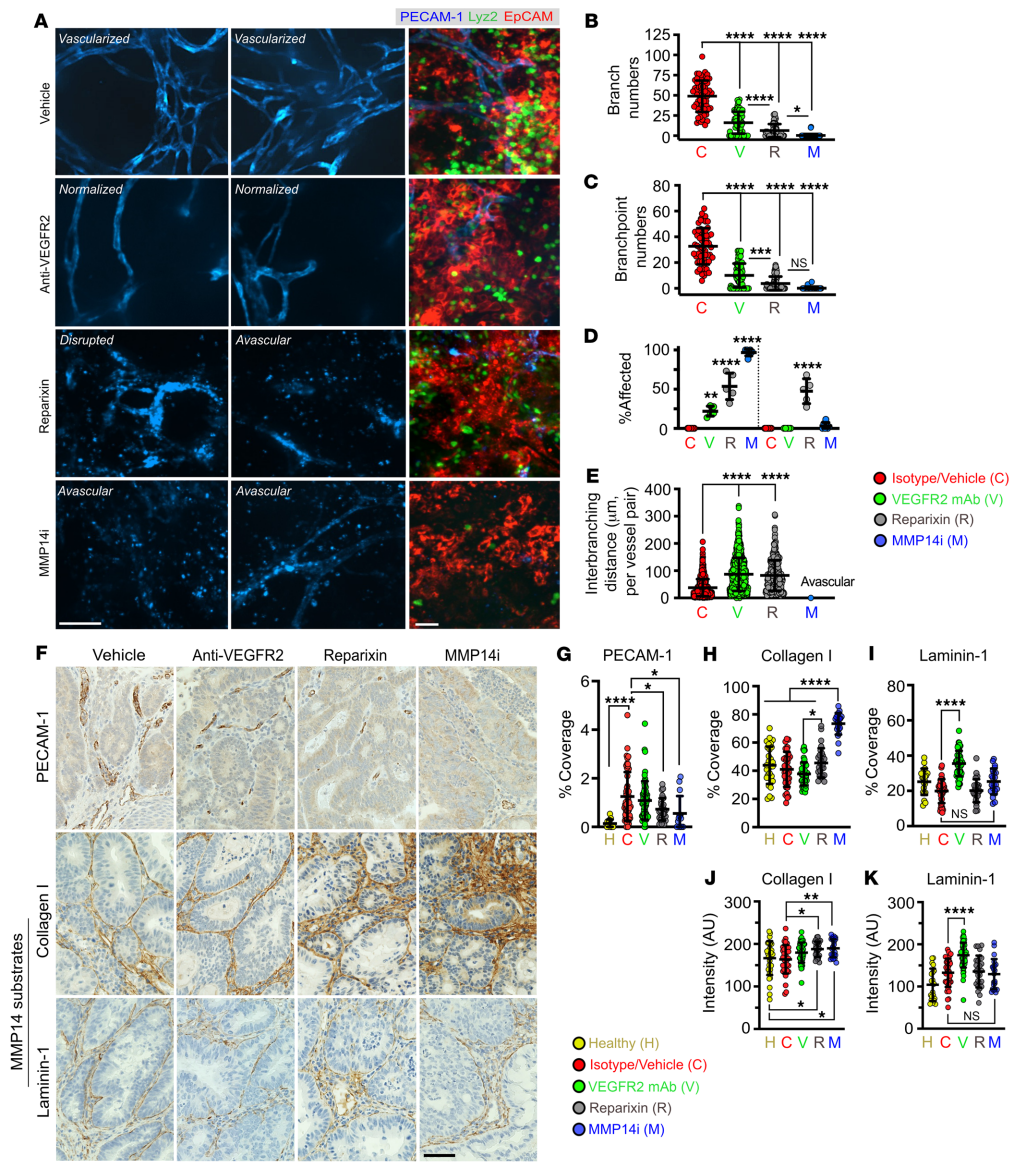
MMP14 activity is required for collagen processing and maintenance of the tumor vasculature. (**A**) Representative whole-mount fluorescence confocal microscopy images of tumor vasculature (stained for PECAM-1, left, middle panels) and of the advanced CRC niche (CRC/EpCAM, red; TANs/Lyz2, green; vessels/PECAM-1, blue, right panel) at treatment endpoints. Scale bars: 50μm. (**B**–**E**) Quantification of vascular architecture parameters from tumor images following specified treatments. Images representative of 3 independent experiments with 50 FOVs analyzed per group (Vehicle/isotype, *n* = 6, Anti-VEGFR2, *n* = 6, Reparixin, *n* = 6, MMP14i, *n* = 8 mice, 1-way ANOVA with Tukey’s multiple comparison test). For analyses presented in panel **L**, a total of approximately 1,000 vessels per treatment conditions were analyzed. *P < 0.05, **P < 0.01, ***P < 0.001, ****P < 0.0001. (**F**) Representative IHC staining of the tumor vascular network (PECAM-1, top), Collagen I (middle, primary substrate) and Laminin-1 (bottom, secondary substrate) for the specified treatment groups. Scale bar: 20μm. (**G**) Quantification of vessel coverage, (**H**) Collagen 1, and (**I**) Laminin 1 coverage in tumor tissues following specified treatments. (**J**) Quantification of staining intensity as an index of Collagen I and (**K**) Laminin-1 levels. For all image analyses, 50 FOVs per treatment group from *n* = 4–5 mice were quantified. *P < 0.05, **P < 0.01, ****P < 0.0001, 1-way ANOVA with Tukey’s multiple comparison test.

Reparixin treatment (5 mg/kg/day), as an alternative to Ly6G-mediated PMN depletion, potently reduced TAN numbers, while slightly decreasing peripheral blood PMNs and monocytes without a significant impact on TAMs (Figure 8C and Supplemental Figure 8, C and D). Importantly, Reparixin treatment phenocopied Ly6G-mediated PMN depletion, reducing tumor burden (Figure 8, D–F), increasing tumor cell death, (Figure 8G with gating shown in Supplemental Figure 8E), and suppressing tumor vascularization (reduced number of branches/branchpoints, elevated avascularity regions, and disrupted vessel morphology, Figure 9, A–D).

MMP14 inhibition by NSC405020 (MMP14i, 2 mg/kg/day) led to a more robust tumor regression (Figure 8, D–G and Supplemental Figure 8, B and E) and decreased tumor vascularization (Figure 9, A–C), resulting in approximately 90% avascularity of tumor tissues but not adjacent regions (Figure 9D).

Intriguingly, although VEGFR2 inhibition induced vessel relaxation by increasing interbranching distances (Figure 9E) and decreased individual and total tumor burden (Figure 8, E and F), interventions by Reparixin or MMP14i were more efficacious in suppressing vascularization (Figure 9, A–D and Supplemental Figure 8F) and reducing tumor burden (Figure 8, D–G) in a PMN-dominant colitis-associated CRC model.

Consistent with Collagen I being a primary substrate of MMP14, tissue deposition of Collagen I (50) but not Laminin-1 (51) (another MMP14 substrate) was significantly enhanced with MMP14 inhibition, resulting in fibrous tumor morphology and reduced vascularization (Figure 9, F–K and Supplemental Figure 8F). Reparixin, but not VEGFR2 treatment, similarly promoted intratumoral Collagen I accumulation, consistent with TANs being the major, though not exclusive, source of MMP14 in the TME.

In the absence of a commercially available small molecule inhibitor to OPN, we attempted to target OPN using BM chimera or antibody-neutralization approaches. Unexpectedly, the majority (approximately 80%) of myeloid OPN^ko/ko^ chimeras (generated by OPN^ko/ko^ BM transfer into irradiated WT hosts) died during the first AOM/DSS cycle due to exaggerated colitis featuring edema, severe weight loss, and colon length shortening (Supplemental Figure 9, A–E). Similarly, antibody-mediated OPN inhibition (clone 103D6 (52), 200 μg, every 48 hours) in an additional colitis model, i.e., IL10^ko/ko^ treated with DSS to expedite disease progression, significantly exaggerated colitis symptoms, promoting severe weight loss, colon shortening, reduced spleen weight, and colitis-related mortality, even at recovery phase (Supplemental Figure 9, F–K). These findings revealed a surprising protective role for OPN in acute and potentially chronic colon injury, indicating that OPN cannot be therapeutically targeted during colitis-driven tumorigenesis.

### MMP14 inhibition curbs tumor development in a CRC PDX model.

**Figure 10:**
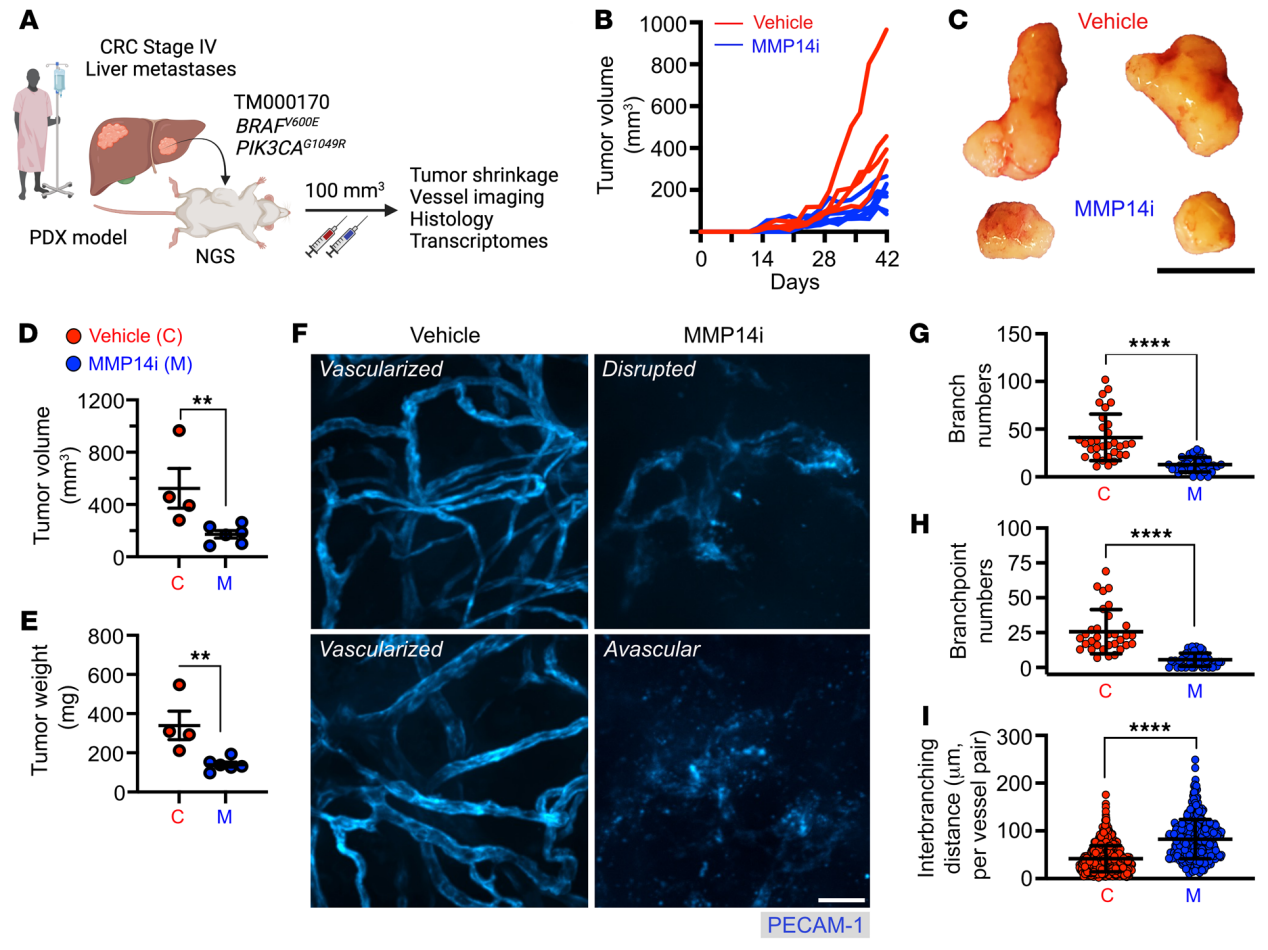
MMP14 inhibition curbs tumor growth in a CRC PDX model. (**A**) Schematic of human CRC tumor grafting into NGS mice, followed by vehicle control (C) or MMP14i treatment (M, NSC405020, 2.0 mg/kg/day (45)). (**B**) Spaghetti plots of individual PDX tumor growth curves and (**C**) Representative endpoint PDX images (day 42/week 6). Scale bar: 1cm. (**D**) Quantification of endpoint tumor burden indexed by volume and (**E**) weight following MMP14 inhibition (vehicle, *n* = 4; MMP14i, *n* = 6 mice). (**F**) Representative whole-mount, fluorescence confocal microscopy images of endpoint PDX vasculature (stained for PECAM-1). Scale bar: 50μm. (**G**–**I**) Quantification of vascular architecture parameters from endpoint tumor vessel images. Images are representative of *n* = 4–6 mice per condition with 50–75 FOVs analyzed per group. For **I**, a total of approximately 1000 vessels per treatment were analyzed. **P < 0.01, ****P < 0.0001, 2-sided student’s *t* test.

**Figure 11:**
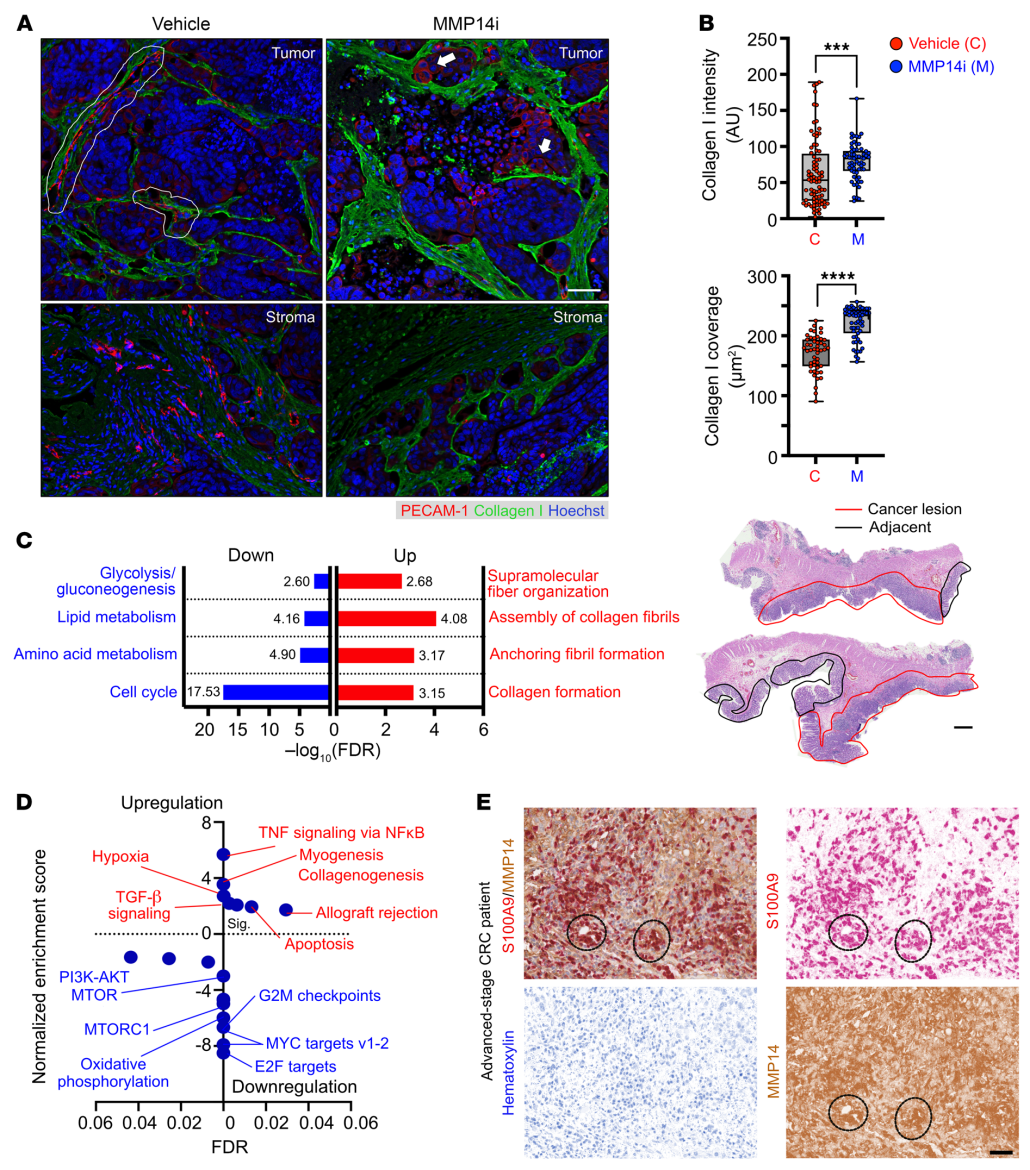
MMP14 inhibition promotes collagen accumulation and profibrotic programs in CRC PDXs. (**A**) Representative fluorescence staining of CRC vasculature (PECAM-1, red) and Collagen I (green, MMP14 substrate) in tumor and stromal regions. Nuclei were counterstained by Hoechst (blue). White outlines and arrows indicate intact and disrupted vessels with abnormal morphologies, respectively. (**B**) Quantification of fluorescence intensity as an index of Collagen I levels (top) and staining coverage in tumor tissue following the specified treatment (vehicle, *n* = 4; MMP14i, *n* = 6 tumors with 50–70 FOVs analyzed per group). ***P < 0.001, ****P < 0.0001, 2-sided student’s *t* test. (**C**) Metascape GO analyses of MMP14i-treated tumors highlight enriched pathways involved in Collagen fibril and matrix formation (red, upregulation) versus cell cycle/metabolism (blue, downregulation). A cutoff of FDR < 0.05 following Benjamini-Hochberg’s correction was set for GO enrichment analysis. (**D**) GSEA of the MSigDB “50 Hallmarks’”gene set specifies pathways upregulated (NES > 0, red) and downregulated (NES < 0, blue) following MMP14i inhibition. Pathways were ranked based on NES with FDR < 0.05 following Benjamini-Hochberg’s correction. (**E**) Representative H&E (top, scale bar: 500μm) and dual-stained IHC images (middle and bottom, scale bar: 30 μm) of advanced-stage CRC patients enriched for MMP14 and MMP14^+^ S100A9^+^ TANs. In H&E view, the cancerous lesions (red outline) and cancer-adjacent colon regions (black outline) of double-core biopsies from a stage III CRC patient were shown. Digital color deconvolution shows separate staining of S100A9 (red), MMP14 (brown), and nuclei (blue) of double-positive TAN clusters (dotted circles) within intratumoral regions.

To support our observations of the MMP14i-mediated suppression of tumor vascularization and growth, we employed an additional, clinically relevant patient-derived xenograft (PDX) model. Stage IV metastatic CRC tumors were grafted into NSG mice, and once tumors were established (approximately 100 mm^3^ in volume) animals were treated with either vehicle control or MMP14 inhibitor (NSC405020, 2.0 mg/kg/day) as illustrated in Figure 10A. NSC405020 treatment had no apparent adverse effects on colon health or on either BM-PMN or tissue PMN counts (Supplemental Figure 9, L–Q). As we have previously established in the AOM/DSS CRC model (1, 2), PMN depletion in PDXs resulted in approximately 2-fold decreases in tumor vascularity and tumor volumes. MMP14i in this model similarly caused a significant tumor regression (reduced tumor volume and weight by over 50%, Figure 10, B–E and Supplemental Figure 10A) and reduced tumor vascularization (Figure 10, F–I), resulting in elevated avascular regions (Supplemental Figure 10E) and robust Collagen I accumulation (Figure 11, A and B). MMP14i treatment exerted minimal impact on systemic and tumor immune cell composition (Supplemental Figure 10, B–D).

To examine the impact of MMP14i on the transcriptional landscape of PDX tumors, mRNA-Seq was performed on excised MMP14i tumors. GO analyses revealed overrepresentation of biological terms involved in negative regulation of vasculature, cellular damage/instability, collagen fiber-fibril assembly, and keratinization-fibrosis (Figure 11C and Supplemental Figure 10, F–H). GSEA using the “50 Hallmarks” human MSigDB data set (26) identified a strong enrichment of pathways associated with collagen formation (*Collagenogenesis* and *Myogenesis*), fibrosis and hypoxia (*TGF-*β *signaling* and *Hypoxia*), and tumor cell death (*Apoptosis* and *Allograft rejection*) in MMP14i-treated tumors (Figure 11D). Thus, functional and transcriptomic assessment of PDX tumors indicated MMP14 as a potential therapeutical target for advanced CRC.

Finally, histological characterization of tissue specimens from patients with grade 3, stage III-IV CRC identified high MMP14 expression localized to cancer lesions and not adjacent colon tissue (Supplemental Figure 11A). Further dual IHC staining for S100A9 and MMP14 followed by digital color deconvolution confirmed an abundant presence of high MMP14-expressing TANs in all 4 patients (Figure 11E and Supplemental Figure 11B), confirming TANs as a major MMP14 source in CRC and establishing the relevance of targeting MMP14 in human disease.

## Discussion

Active UC and CRC lesions feature robust PMN/TAN accumulation (1–4, 16), suggesting that these cells play important roles in inflammation and CRC progression. Although, in the past, PMNs were considered terminally differentiated phagocytes, the cellular plasticity and the malleable transcriptional and functional nature of PMNs and TANs are increasingly recognized (11, 12, 14, 15). Here, we examined the extent to which disease-specific transitional states (colitis to CRC transition) and spatial separation of circulating versus tissue PMNs affect PMN/TAN transcriptional and functional programs.

Given the relatively low PMN/TAN transcriptional activity (53, 54), to achieve a reliable sequencing depth of PMN transcriptomes compared with single-cell RNA-Seq, FACS-sorted PMN/TAN transcriptomes were analyzed by total-input bulk RNA-Seq (15). Such profiling revealed a distinct spatial separation of tissue and blood PMNs and TANs regardless of the disease state, confirming transcriptional adaptability and a niche-specific impact. Omics approaches complemented by in vivo and in vitro functional studies revealed that TANs undergo proangiogenic specialization within the CRC niche, driving tumor vascularization and shaping vascular architecture. Our studies identified MMP14 and OPN to be robustly induced in TANs, serving nonoverlapping roles in mediating the proangiogenic TAN function. Both factors have been previously associated with angiogenesis (32, 33, 35, 39, 44); however, we now established TANs as a major MMP14/OPN source in CRC tumors and demonstrated a specific role for each of these molecules in driving tumor vascularization via a VEGF-independent route. Thus, our findings highlight the emerging complexity of the immune-associated angiogenic drivers in the tumor microenvironment, adding new elements to the already established TAN-derived proangiogenic factors including MMP-9, VEGF, FGF-2, and Bv8/S100 proteins (summarized in a recent review) (55). The robust impact of MMPs on tumorigenesis outcomes suggest the potential utilization of natural or synthetic/modified tissue inhibitors of metalloproteinases (TIMPs) as potential anticancer therapeutic. Of note, we identified TANs as a major but not the only source of MMP14 and OPN in the tumor microenvironment, and TAN specific contribution compared with contributions of other cells, including ECs or TAMs, remains to be determined using cell-specific knockout approaches. Similarly, the underlying mechanisms driving TAN specialization in the tumor niche and the induction of MMP14/OPN are not well defined, but may involve activation of the TGF-β pathway, which has been shown to regulate OPN expression (56, 57) and synergize with MMP14 to control vessel stability (58, 59). Some of these aspects are being actively explored in our lab.

Whole-mount tumor imaging and 3D reconstruction analyses revealed close TAN association with tumor vasculature, consistent with the idea of TANs guiding tumor vascular network formation. Meanwhile, antibody-mediated TAN depletion or small molecule-inhibition of TAN tumor infiltration significantly impaired tumor vascularization and vessel depth penetration in both AOM/DSS and PDX CRC models. This was supported by in silico analyses and mining of published CRC patient data sets, revealing significant correlation between TAN markers (*S100A8*, *S100A9, SPP1,* and *MMP14*) and established EC genes (*PECAM1*, *VWF*, and *CDH5*).

Mechanistic ex vivo studies identified TANs to actively promote EC migration and spontaneous vessel sprouting and branching via the release and respective actions of OPN and MMP14. MMP14 remodels the extracellular matrix by degrading collagens, fibronectin, and laminin (45, 50, 51, 60, 61), facilitating vascular network expansion (62–65). Complementarily, OPN can activate PI3K/AKT and ERK1/2 pathways in ECs to enhance vessel tip migration and induce angiogenesis by activating the avβ3/PI3K-AKT/eNOS/NO signaling in EC progenitors (33). In vivo inhibition of MMP14 by NSC405020 led to aberrant tissue collagen I accumulation, resulting in highly fibrotic tumors, which significantly regressed with treatment. NSC405020 targets the PEX allosteric domain of MMP14, compromising its dimerization and collagen cleavage (45, 61). Interestingly, while MMP14i increased tumor Collagen I levels, it did not significantly impact the levels of Laminin-1, suggesting a lower MMP14 affinity toward Laminin-1 and/or reduced TAN accessibility to Laminin-rich regions, such as the tumor basement membrane. Supporting the idea that MMP14 inhibition–driven induction of fibrosis led to the observed tumor regression, genetic deletion of MMP14, or allosteric inhibition of the MMP14 PEX domain has been shown to impair collagen degradation (66, 67), limiting tumor growth (45).

Overrepresentation of pathways related to collagenogenesis and fibrosis were similarly noted by transcriptomic analyses of MMP14i-treated PDX tumors. Increased collagen deposition leading to ECM stiffening can also impair EC junction integrity (68), indicating that TANs, via the release of MMP14, create a more permissive environment for angiogenic vessel sprouting in developing CRC tumors.

Finally, our work has established what we believe to be a novel therapeutic framework for CRC, focused on the inhibition of TAN angiogenic properties. VEGF/VEGFR signaling is considered a classical driver of angiogenesis, guiding formation of vascular networks in developing tumors (49, 69). In fact, PMNs have been shown to produce VEGF on their own, promoting angiogenesis under certain conditions (70, 71). Numerous clinical trials (NCT04547205, NCT02942329, and NCT03494231) and VEGFR-targeting drugs showed promise in anticancer therapy and are now under development (49, 72, 73). Interestingly, although, as expected, VEGFR2 inhibition led to normalization of tumor vasculature and overall reduction in vascular density, we showed that such treatment was less effective in inducing tumor regression compared with MMP14i, which more robustly and reproducibly disrupted tumor vasculature and suppressed tumor growth in both AOM/DSS and PDX models. MMP14 inhibition was specific to antitumoral response and did not exaggerate colitis symptoms, as was the case with OPN inhibition. Mechanisms underlying the protective OPN function in colitis are not known; however, given the severe exacerbation of colitis disease with OPN treatment, these data argue against utilization of such therapy for inflammation-driven tumors such as CRC.

In summary, our work revealed proangiogenic TAN reprograming by the CRC niche, resulting in MMP14/OPN-dependent promotion of tumor vascularization. Our data further demonstrate that targeting the pro-angiogenic TAN activity and specific MMP14 inhibition may provide promising therapeutic option to suppress tumor growth.

## Methods

### Animals.

Considering sex as a biological variable, all animal studies included equal representation of male and female mice. WT C57BL6J, OPN KO (B6.129S Cg-Spp1^tm1Blh^/J, no. 004936) purchased from Jackson Laboratory and Lyz2^EGFP^ reporter mice (Lyz2tm1.1Graf, gift from Harris R. Perlman, Northwestern University) were maintained under specific pathogen-free conditions at Northwestern University animal facilities. PDX-bearing (TM00170) NSG mice (NOD.Cg-Prkdcscid Il2rgtm-1Wjl/SzJ) were purchased from Jackson Laboratory.

### AOM/DSS colitis-induced CRC model.

Tumor formation was induced by administration of a single dose of azoxymethane (AOM, 12.5 mg/kg i.p.) followed by 3 to 5 cycles of 3% DSS (1 cycle = 5 days of DSS + 14 days recovery) (1, 24). Colitis ulcerations and CRC lesion formation (early, low-grade, 3 cycles and advanced poorly differentiated CRC tumors, 5 cycles) was monitored biweekly by high-resolution endoscopic imaging. Total number of CRC tumors per colon was quantified and defined as tumor burden, and individual tumor volumes were calculated as volume = length × width^2^ × ½. PMN depletion in this model was initiated and maintained by repeated administration of increased dosing of rat anti-Ly6G mAb (clone 1A8, i.p., daily, 25–100 μg) followed by secondary anti-rat IgG antibody (MAR18.5, 50 μg, every 48 hours) (27). For inhibition studies, CRC-bearing mice were injected i.p. with either Reparixin (5.0 mg/kg/injection, daily) (46, 47), NSC405020 (2.0 mg/kg/injection (45), 3 times per week), or anti-VEGFR2 monoclonal antibody (50 mg/kg/injection, 3 times per weeks, clone DC101) (49) at the start of cycle 4 (week 11) where developing tumors transition toward advanced, high-grade CRC. Drug concentrations were selected based on previously published studies.

### Tumor cell dissociation and FACS sorting.

Excised tumors were digested by collagenase type VIII (1 mg/mL), hyaluronidase (1 mg/mL), and DNAse I (100 μg/mL) solution. Single-cell suspensions were generated by 0.05% Trypsin treatment (10–15 minutes, 37°C) and assessed for viability by Trypan Blue staining. FACS sorting of PMNs was performed on a 6-color laser FACS Aria sorter. EpCAM^+^ tumor/epithelial cells and Ly6G^neg^ CD64^hi^ macrophages were gated out to avoid RNA contamination. CD45^+^/CD11b^+^/Ly6C^int^/Lyz2EGFP/Ly6G^hi/int^ PMNs/TANs were sorted into RNA lysis buffer supplemented with RNaseOUT (10 U/μL).

### Tissue/cell analyses.

Tissue IHC, gene expression analysis by quantitative RT-PCR, flow cytometry, and immunofluorescence were performed as detailed in the supplemental material.

### Whole-mount spinning-disk confocal microscopy.

For tumor/colon tissue imaging colons were cut open longitudinally to expose the lumen, washed to remove fecal matter and mucus, secured by Vetbond tissue adhesive onto a silicon pedestal and submerged into 37°C-warmed phenol red–free DMEM. Nonblocking anti-PECAM-1 antibody (CBL1337) conjugated to DyLight-650 NHS Ester administered via Retroorbital injection 30 minutes prior to imaging and EpCAM antibody (clone G8.8, 118205) conjugated with PE or FITC stained in situ were used to visualize blood vessels and tumor cells, respectively. All imaging was performed on UltraVIEW VoX imaging system built on an Olympus BX-51WI Fixed Stage illuminator equipped with a Yokogawa CSU-X1-A1 spinning disk, a Hamamatsu EMCCD C9100-50 camera and a Modular Laser System with solid state diode lasers with DPPS modules for 488, 561, and 640 nm and appropriate filters (Perkin Elmer). Data analysis was performed using ImageJ and Imaris software.

### RNA-Seq and bioinformatic analysis.

Bulk RNA-Seq of FACS-sorted tumor/colon tissue PMNs was performed and analyzed following rigorous quality checks per published methodology as detailed in the supplemental material. Low-input total cDNA library construction and RNA-Seq was conducted at the Northwestern University sequencing core facility using Illumina HiSeq 4000 NGS or the Illumina NovaSeq 6000 systems (Illumina). GSEA and GO Enrichment Analysis were performed using the HTSAnalyzeR package with a *P* value cutoff of 0.05 after Benjamini-Hochberg’s correction. For correlation analyses of vessel and immune markers, gene expression CRC patient data sets published in TCGA were accessed through cBioPortal (28) for Cancer Genomics (https://www.cbioportal.org/). CRC patient cohort RNA-Seq data (*n* = 594, TCGA PanCancer Atlas) was accessed using keywords “bowel” and “colorectal adenocarcinoma”.

### Statistics.

Statistical differences for 2 groups comparisons were determined by 2-tailed unpaired student’s *t* test for normally distributed data and Mann-Whitney U test for nonparametric data. For 3 or more groups, 1-way ANOVA followed by Dunnett’s or Tukey’s multiple comparison was used. DEGs in bulk RNA-Seq experiments were analyzed by 2-sided *t* test with Bayes moderation (eBayes function in R/bioconductor limma package) (74). All data, unless otherwise stated, are shown as ± SEM.

### Study approval.

For expression analyses, freshly frozen resected human CRC tissue was obtained from the Northwestern University-Robert H. Lurie Comprehensive Cancer Center Pathology Core Facility with approval by and in accordance with Northwestern University IRB protocol (STU00212299). All experimental protocols involving animals were approved by the Northwestern University Institutional Animal Care and Use Committee (PHS assurance number A328301).

### Data availability.

All original files for RNA-Seq data were submitted to NCBI’s Gene Expression Omnibus database with the accession number GSE232217. The Supporting Data Values file contains all data points shown in graphs.

## Author contributions

TMB and RS conceived designed and conducted experiments. TMB, LKY, EN, JMU, JAD, XR, KA and CJH performed downstream in vitro biochemical, histological, and functional analyses. BW and MJS performed sequencing and ran initial processing and enrichment analyses of RNA-Seq data sets. TMB performed downstream bioinformatic analyses. SBH, GYY, PSD, and YSV provided clinical biospecimens and supervised histological analyses. DPS assisted with the analysis of tumor vessel imaging and performed bone-marrow chimera experiments. SBH, SML, and RS contributed reagents, materials, or analysis tools. TMB and RS wrote the manuscript. All authors edited the manuscript.

## Supplementary Material

Supplemental data

Supporting data values

## Version 1 02/08/2024

In-Press Preview

## Version 2 04/01/2024

Electronic publication
