# Supplementary material for: Tissue-specific reprogramming leads to angiogenic neutrophil specialization and tumor vascularization in colorectal cancer
Source: J Clin Invest. 2024 Apr 1;134(7):e174545. doi: 10.1172/JCI174545 (PMC10977994; doi:10.1172/JCI174545)
Supplement: Supplemental data [file jci-134-174545-s055.pdf]

Tissue-specific reprogramming leads to angiogenic neutrophil specialization and tumor vascularization in colorectal cancer

Authors: Triet M. Bui[1,6], Lenore K. Yalom[1], Edward Ning[1], Jessica M. Urbanczyk[1], Xingsheng Ren[1], Caroline J. Herrnreiter[1], Jackson A. Disario[1], Brian Wray[2], Matthew J. Schipma[2], Yuri S. Velichko[3], David P. Sullivan[1], Koki Abe[4], Shannon M. Lauberth[4], Guang-Yu Yang[1], Parambir S. Dulai[5], Stephen B. Hanauer[5], Ronen Sumagin[1].

Affiliations: [1] Department of Pathology, Northwestern University Feinberg School of Medicine, 300 East Superior St. Chicago, IL 60611. [2] Quantitative Data Science Core, Lurie Cancer Center, Northwestern University Feinberg School of Medicine, 750 N. Lake Shore Drive, Chicago, IL 60611. [3] Department of Radiology Northwestern University, Feinberg School of Medicine, 676 North St. Clair Street, Chicago, IL 60611. [4] Simpson Querrey Institute for Epigenetics and Department of Biochemistry and Molecular Genetics, Feinberg School of Medicine, Northwestern University, Chicago, IL 60611, USA. [5] Department of Medicine, Gastroenterology and Hepatology, Northwestern Memorial Hospital, 259 E Erie St, IL 60611. [6] Present Address: Department of Medical Oncology, Dana-Farber Cancer Institute, 450 Brookline Ave, Boston, MA 02215.

Supplemental Materials and Methods

Animals: Wildtype C57BL6J, OPN KO mice (B6.129S Cg-Spp1tm1Blh/J, Strain #004936), purchased from Jackson Laboratory (Bar Harbor, ME) and Lyz2EGFP reporter mice (Lyz2tm1.1Graf, gift from Dr. Perlman, Northwestern University) ages 8–16 weeks, were maintained under specific pathogen-free conditions at Northwestern University, Feinberg School of Medicine animal facilities. Patient-derived xenografts (PDX, TM00170) in NSG mice (NOD.Cg-Prkdc-scid Il2rgtm-1Wjl/SzJ) were obtained from Jackson Laboratory (Bar Harbor, ME). At the end of all experimental procedures, animals were euthanized via rapid cervical dislocation. All experimental protocols were approved by the Northwestern University Institutional Animal Care and Use Committee (IACUC).

Cell culture: Mouse microvascular brain endothelial cell (bEnd.3, ATCC, Manassas, VA) were grown in Dulbecco's modified Eagle's medium (DMEM) supplemented with 10% fetal bovine serum (FBS), 1% L-glutamine, and 1% penicillin-streptomycin. Human umbilical endothelial cells (HUVECs, ATCC) were grown in human endothelial SFM supplemented with 1% L-glutamine, endothelial cell growth supplement (0.0375 mg/mL), 10% FBS (Atlanta Biologicals, Flowery Branch, GA), 1% penicillin-streptomycin, and 1% non-essential amino acids in a humidified atmosphere at 37°C with 20% O_2 and 5% CO_2. Mycoplasma was frequently tested in these cell lines by PlasmoTest™ Detection Kit (Invitrogen, Waltham, MA).

Recombinant proteins and monoclonal antibodies: Monoclonal antibodies targeting murine (clone LEM-2/15.8, MA3328) and human/murine MMP14/MT1-MMP (clone EP1264Y, ab51074) were purchase from EMD Millipore (Burlington, MA) and Abcam (Cambridge, MA). Murine recombinant MMP14/MT1-MMP (45.9 kDa, LS-G14692, LSBio,

Seattle, WA) were reconstituted in a solution of 20mM Tris, 150mM NaCl, pH8.0 at stock concentration of 100 µg/mL and used for cell-based assays. Full-length (60 Da, 918-MPN, R&D Systems, Minneapolis, MN) and pro-/catalytic domain (31kDa, 918-MP-010, R&D Systems) of human recombinant MMP14/MT1-MMP were reconstituted to stock concentration of 440 and 220µg/mL for cell-based assays. Monoclonal antibodies targeting murine (clone AF808, R&D Systems) and human SPP1/OPN (clone 1B20, NB110-890620, Novus Biologicals, Littleton, CO) were reconstituted to stock concentrations of 200µg/mL and 1mg/mL, respectively prior to use. Murine (VEGF164 fragments, 493-MV-CF, R&D Systems) and human recombinant VEGF (293-VE-CF) were reconstituted at 50 and 100µg/mL, respectively, for cell-based assays. Monoclonal antibodies targeting murine VEGF (clone A-20, sc-152, Santa Cruz, Dallas, TX), human/murine S100A9 (NB100-89726, Novus Biologicals), murine CD31/PECAM-1 (clone Mec13.3, CM303, Biocare, Concord, CA), murine/human Collagen I (1310-01, SouthernBiotech, Birmingham, AL), murine/human Laminin-1 (L9393, Sigma Alrich, St. Louis, MO) were used for immunohistochemical (IHC) staining, unless otherwise stated. The following antibodies were used for immunophenotyping by flow cytometry: anti-Ly6G (clone 1A8), anti-CX3CR1 (clone SA011F11), and anti-CD11b (clone M1/70) from BD Biosciences (San Jose, CA); anti-Ly6C (clone HK1.4), anti-CD45 (clone 30-F11), anti-CD64 (clone X54–5/7.1), anti-EpCAM (clone G8.8), anti-CD114/CSF3R (clone LMM741), anti-CD24 (clone 30-F1), anti-Gr1 (clone RB6-8C5), anti-CXCR2 (clone SA044G4), anti-CD62L (clone MEL-14), anti-IL17A (clone TC11-18H10.1), anti-CD9 (clone MZ3), anti-CD117/c-Kit (clone 2B8), anti-CD44 (clone IM7), anti-CD14 (clone Sa14-2), anti-CD80 (clone 16-10A1), anti-CD101 (clone Moushi101) from Invitrogen.

Small-molecule inhibitors and treatment dosage for *in vivo* inhibition studies: CXCR2 inhibitor (Reparixin, Cat: A12383, CAS No. 266359-83-5, AdooQ Bioscience, Irvine CA), broad-spectrum MMP inhibitor (GM6001, Cat: A13320-10mM-DMSO, CAS No. 14280-36-2, AdooQ Bioscience) and allosteric inhibitor of PEX domain of MMP14/MT1-MMP (NSC 405020 or compound 9, CAS No. 797-07-6, Cat:444295 Sigma Alrich) were dissolved in dimethyl sulfoxide (DMSO) at stock concentration of 5mmol/L, 10mmol/L, and 3.84mmol/L, respectively. For AOM/DSS and PDX CRC models, *in vivo* solvents of Reparixin and NSC 405020 were made freshly with provided formulation from manufacturers (1% DMSO, 30% PEG300, 1% Tween 80) and were administered at dosage of 5.0 mg/kg/injection (1, 2) (daily) and 2.0 mg/kg/injection (3) (3 times per week), respectively, at the indicated timepoints.

Colitis-induced CRC model by AOM/DSS: To initiate carcinogenesis, mice were given a single dose of azoxymethane (AOM) by intraperitoneal (i.p.) injection (12.5 mg/kg body weight) (4, 5). A week after, mice were treated with up to 5 cycles of DSS 3% in drinking water (1 cycle = 5 days of DSS + 14 days recovery). The presence of colitis ulcerations and CRC lesions was monitored biweekly by high-resolution endoscopic imaging. Based on previously published phenotypic examination and histological scoring of CRC tumors by board-certified pathologists, we established that early tumors formed after 2-3 cycles of DSS at week 7-8 and were classified as well-differentiated (low-grade), while advanced and poorly-differentiated CRC tumors formed only after more than 4 cycles of DSS at week 13-15. Animals were sacrificed either at the time of acute colitis (1 cycle of DSS), or upon confirmation of low-grade tumors (3 cycles of DSS, weeks 7-8), or once high-grade tumors were formed (5 cycles of DSS, 15 weeks). Immediately after sacrifice,

peripheral blood was drawn from the vena cava; bone marrows from both hind-legs (femur and tibia) were collected; spleen and tumor-bearing colons were extracted for phenotypic evaluations and downstream molecular/functional assays. The total number of CRC tumors per colon was quantified and defined as tumor burden, and individual tumor volumes were mathematically quantified as Volume = length × width^2 × ½. For this specific model, temporary PMN depletion was initiated and maintained at indicated timepoints by repeated administration of incremental doses of rat anti-Ly6G mAb (clone 1A8, i.p., daily, 25-100μg, BioXcell, Lebanon, NH) followed by secondary conjugation of anti-rat IgG antibody (MAR18.5, 50μg, every 48 hours, BioXcell, Lebanon, NH) (6). The administration of anti-Ly6G mAb and anti-rat MAR18.5 was maintained throughout one cycle of DSS (21 days) to established PMN-depleted CRC tumors with an effective reduction in tumor-infiltrating PMNs confirmed by IHC and whole-mount confocal imaging (**Supplemental Figure 5A-B**, previously performed in (5)). For *in vivo* inhibition studies, CRC-bearing mice were injected i.p. with either Reparixin (5.0mg/kg/injection, daily (1, 2)), NSC 405020 (2.0mg/kg/injection (3), 3 times per week), or anti-VEGFR2 monoclonal antibody (50mg/kg/injection, 3 times per weeks, clone DC101 (7), BE0060, BioXcell, Lebanon, NH) at the start of cycle 4 (week 11) where developing tumors transition towards advanced, high-grade CRC. Drug concentrations were selected based on previously published studies.

Patient-derived Xenograft (PDX) Model of Colorectal Cancer: For PDX experiments, a total of 15 PDX-bearing mice were purchased from Jackson Laboratory (Bar Harbor, ME). PDXs were established by the Jackson Labs as follows; metastatic stage IV colon adenocarcinoma with BRAF^{V600E} and PI3KCA^{G1049R} mutations derived from a 30-year-old

male patient (Colon Cancer, PDX Model TM00170) were subcutaneously grafted ($2x10^6$ tumor cells/mouse in 40µL PBS) into flanks of NOD.Cg-Prkdc-scid Il2rgtm-1Wjl/SzJ NSG mice aged 7-8 weeks (strain 005557 NSG). Through the duration of the experiments, tumor diameters were measured bi-weekly by calipers and size was calculated using the mathematical formula: Volume = $4\pi/3 \times (\text{length} \times \text{width} \times \text{height}) \times 1/8$. Tumor volumes and corresponding growth curves were statistically analyzed using Mann-Whitney's U test in GraphPad Prism. Once tumors reached $100mm^3$, mice were randomly divided into 2 treatment groups (Isotype control and NSC405020). NSC405020 was administered 1.0mg/kg/mice (3 times per week, i.p.) for 21 days (endpoint). Doses of inhibitors were selected based on previous work (3, 9). Following sacrifice, tumors were measured, weighed, excised and split into three portions for the following analyses: (i) whole-mounted and imaged by spinning-disk confocal microscopy for tumor vasculature structure, (ii) fixed in 4% PFA and embedded in paraffin blocks for IHC, and (iii) immediately dissociated into a single-cell suspension for flow cytometric and downstream functional analyses. For all mice peripheral blood was collected from vena cava, and red blood cells were lysed by ACK Lysing Buffer (Gibco, Life Technologies), spleen and bone marrow were harvested for immunophenotyping and PMN/CRC co-culture assays as indicated in AOM/DSS-induced CRC experiments.

Tumor single-cell dissociation: At experimental endpoints, mice were sacrificed using isoflurane-induced anesthesia followed by cervical dislocation. For the AOM/DSS model, excised colons were opened longitudinally to expose the luminal surface and tumors were resected using stereo-dissecting microscope. The extracted tumors were incubated in the dissociation solution of collagenase type VIII (1mg/mL, Sigma Alrich), hyaluronidase

(1mg/mL, Sigma Alrich) and DNAse I (100µg/mL, Invitrogen) in Dulbecco's modified Eagle's medium supplemented with 10% FBS (Atlanta Biologicals) with shaking motions at 37°C for 45-60 minutes. Tumor cell suspensions were washed with PBS and further digested by 0.05% Trypsin (10-15 minutes, 37°C), and resulting single-cell suspension was filtered through a 70-µm strainer three times. Live single cells were counted using Trypan Blue and hemocytometer prior to downstream assays.

Flow cytometry and fluorescence-activated cell sorting (FACS) of PMNs and myeloid cells: Following blood and tissue extraction, single-cell suspensions of immune and/or cancer cells were double-filtered using 70-µm cell strainers (Corning, Corning, NY), Fc-blocked (anti-mouse CD16/32, clone 93) and immunostained with indicated fluorophore-conjugated flow antibodies or with non-conjugated antibodies (when stated) followed by staining with goat anti-mouse (A21050, Life Technologies, Carlsbad, CA) or donkey anti-rabbit (A31573, Life Technologies) secondary antibodies. Viability Dye eFluor 506 (Thermo Scientific) was used to gate out dead cells. Stained cells were analyzed using BD LSRFortessa flow cytometer and compensated using beads conjugated with single-color antibody corresponding to each color channel. Appropriate Fluorescence-Minus-One (FMO) controls were used as gating control for cell population intensity analyses. Analysis was performed on FlowJo V10 software (Treestar, Ashland, OR). For intracellular staining of angiogenic factors, cells were gently fixed with PFA 4% and permeabilized using BD Cytofix/Cytoperm followed by BD Perm/Wash buffer (Fixation/Permeabilization Kit, 51-2091KZ, BD Bioscience). Cells were stained with primary antibodies of interest overnight at 4°C, blocked with 10% normal goat serum for

1 hour prior to secondary staining by goat anti-mouse or donkey anti-rabbit antibodies for 2-3 hours.

For sorting of PMNs or other myeloid cells, we utilized Lyz2^{EGFP} reporter mice with additional staining for Ly6G, Ly6C, CD11b, CD45, and CD64. FACS was performed on a 6-color laser sorter FACS Aria at the Northwestern Flow Cytometry core. EpCAM^{+} tumor or epithelial cells as well as Ly6G^{neg} CD64^{hi} macrophages were gated out to avoid RNA contamination from other cell types. CD45^{+}CD11b^{+}Ly6C^{int} Lyz2^{EGFP}Ly6G$^{hi/int}$ were defined as PMNs or TANs and sorted directly into RNA Lysis Buffer (RLT plus buffer, Qiagen), supplemented with excess amount of RNaseOUT (10U/µL, Invitrogen). RNA from PMNs was immediately extracted using the RNeasy Mini Plus Kit (Qiagen) and subjected to RNA integrity (RIN) analysis. For quality control of PMN cell sorting, additional replicates were performed with identical gating strategy and subjected to either more rigorous flow cytometric analyses using additional PMN markers or using Cytospin and immunofluorescence for imaging. Enrichment of PMN gene sets was performed following RNA sequencing to confirm the transcriptomic identity of the sorted cells as well as validity of downstream bioinformatic analyses (see **Supplemental Figure 2**).

Immunohistochemistry: All histology services were provided by the Northwestern University Mouse Histology and Phenotyping Laboratory (MHPL) which is supported by NCI P30-CA060553 awarded to the Robert H Lurie Comprehensive Cancer Center. For murine tumor and adjacent colon tissue, samples were fixed with 4% PFA, paraffin-embedded and 5µm sections mounted onto Apex Superior Adhesive Slides (Leica, Wetzlar, Germany) were incubated at 60°C overnight before deparaffinization in xylene and serial alcohol immersion. For S100A9, Osteopontin (OPN), PECAM-1, Laminin-1,

and Collagen I antibodies, epitope retrieval was carried out with sodium citrate buffer pH 6.0 at 110^oC for 10-20 minutes. For MMP14, epitope retrieval was carried out in pressure cooker with EDTA buffer pH 9.0 at 110^oC for 10-20 minutes. Slides were stained using Dako Autostainer Plus (Dako Omnis; Agilent,) by performing sequential blocking with H_2O_2 and Dako envision kit Protein Block (Dako Omnis), 90 minutes incubation with primary antibodies (S100A9, 1:3000 dilution; PECAM-1/CD31, 1:500; MMP14, 1:400 dilution; OPN, 1:1000 dilution; Collagen 1, 1:750 dilution; Laminin-1, 1:1000) followed by 30 minutes incubation with secondary antibodies (Mach2 Rabbit HRP-polymer, #rhrp520 or Jackson ImmunoResearch, #705-065-147) and counterstaining with Hematoxylin (#245-651, Fisher Scientific). H&E staining was performed by an automated staining platform Leica Autostainer XL with reagents including Hematoxylin (#245-651, Fisher Scientific) and Eosin (#3801600, Leica Surgipath).

Human CRC tissue double staining was performed by sequential overnight primary (anti-MMP14, 1:200, 90 minutes) and anti-rabbit secondary antibody (Mach2 Rabbit HRP-polymer, 30minute) incubation, detected using Bond Polymer Refine detection (cat#: DS9800, Leica Biosystem), followed by overnight primary anti-S100A9 (1:4000, Novus Biologicals NBP3-07983) and 30-minute anti-mouse secondary antibody incubation and detected using Bond Polymer Refine Red Detection (cat#: DS9390, Leica Biosystem).

All slides were scanned using a NanoZoomer 2.0-HT slide scanner and image analyses was performed by NDP.view2 Viewing software (Hamamatsu Photonics, Hamamatsu, Japan) and ImageJ (NIH, Bethesda, MD). Double IHC images were color deconvoluted to separate channels (using Vector for Fast Blue-Fast Red-DAB, ImageJ).

Gene expression analysis by quantitative RT-PCR: For human IBD and CRC specimens, fresh and unidentified tissues were obtained under the approved IRB (STU00214234) in collaboration with Dr. Stephen Hanauer, operated within the main bio-repository IRB (STU00203172). Resected tissue was gently washed in PBS supplemented with excess RNaseOUT (10U/µL), minced, and digested using solution of Liberase (collagenase I/II, 25µg/mL from a stock of 2.5mg/mL, Roche Diagnostics, Indianapolis, IN) and DNase I (0.5U/mL, grade II, Roche Diagnostics, Indianapolis, IN) by three rounds of 10-min intervals of continuous rotation (37°C, 800rpm). Single cell suspensions were double-filtered through 70-µm strainers, collected in 4% BSA-coated 50mL tubes, pelleted, and lysed in RNA Lysis Buffer provided in the RNeasy Mini Plus Kit (74034, Qiagen, Germantown, MD). For mouse tissues and cultured cells, total RNA was extracted using the RNeasy Mini Plus Kit with one-step removal of genomic DNA by gDNA Eliminator Mini-spin columns per manufacturer instructions (#74034, Qiagen, Germantown, MD). The extracted RNA was measured by a ND-1000 Spectro-photometer (NanoDrop Technologies, Wilmington, DE) where Abs_{260}/Abs_{280} ratio was used as an indicator of RNA purity. Complementary DNA (cDNA) was then synthesized from 1 to 2µg of RNA by reverse transcription with random primers using Applied Biosciences cDNA Synthesis Kit (Thermo Scientific, Waltham, MA). Quantitative real-time PCR was performed on 100ng of cDNA in a SYBR Green Master Mix (Roche Diagnostic) using Applied Biosystem Quant-studio 3 Real-Time PCR (Thermo Fisher) with 40-50 cycles of amplification depending on the available yield of cDNA. The CT values were normalized to the mean of four housekeeping genes including GAPDH, β-actin, RPL13A, and β2-microglobulin. Relative expression data were analyzed using the $2^{-\Delta\Delta CT}$ method. A

change in the expression of the genes of interest was considered biologically significant if the fold-change was greater than 1.5 or less than 0.5. Analysis of each gene was performed at least in duplicates using primers purchased from IDT (Coralville, IA). Primer sequences are available on contact with the corresponding author.

Bone Marrow Transplantation of OPN$^{ko/ko}$ Chimera & Acute Colitis Model with Antibody-based OPN Depletion.

Bone marrow (BM) chimeras were generated and maintained using a standard protocol as previously described (10, 11). Bone marrow donors and recipients were always matched according to age and sex. Briefly, 10-week-old wild-type mice were lethally irradiated with a 1000-cGy dose using a Gammacell 40 Exactor ^{137}Cs irradiator (Best Theratronics, Ottawa, ON, Canada) and reconstituted with BM cells (15-20 million) from 8-week-old B6.129S Cg-Spp1^{tm1Blh} (OPN$^{ko/ko}$ donors) or wildtype donors C57BL/6 (n=10) 24 hours after whole-body irradiation. As a result, we generated OPN$^{ko/ko}$ $\rightarrow$ WT recipients (n=15) and 'OPN$^{wt/wt}$ $\rightarrow$ WT recipients (n=10) in preparation for AOM/DSS colitis-induced CRC regimen. Drinking water of chimera mice was supplemented with sulfamethoxazole and trimethoprim suspension for 4 weeks to prevent opportunistic infection. Following 4 weeks of antibiotics, fecal matters from healthy mice were transferred to the cages of chimera to supplement them with healthy microbiomes for 2 more weeks. Chimeric mice were allowed for BM reconstitution for 6 weeks prior to a single AOM injection (12.5mg/kg) and 3-5 cycle of DSS (3% w/vw). Unexpectedly, 80% OPN$^{ko/ko}$ chimera were unable to survive the first cycle of DSS leading to early termination of the study and re-assessment of disease indexes and colon phenotypes. To further confirm the disease phenotypes observed in OPN$^{ko/ko}$ chimera, acute colitis experiment was performed in IL10$^{ko/ko}$ mice

(B6.129P2-Il10^{tm1Cgn}/J) that were continuously injected with anti-OPN mAb (clone 103D6 (8), 200μg/injection, 3 times per week) or with MMP14 inhibitor NSC 405020 (3) (1 mg/kg/injection, 3 times per week), or with isotype control (200μg/injection, 3 times per week). Survival and disease activity index (DAI) was assessed during 6 days of DSS and 11 days of recovery.

Low-input total cDNA library construction and Illumina sequencing: RNA-seq was conducted at the NUSeq Core Facility. Total RNA was extracted from FACS-sorted immune cells using RNeasy Plus Mini Kit (Qiagen), followed by quantification with Qubit and RIN quality assessment with 2100 Agilent Bioanalyzer (Agilent Technologies, Santa Clara. CA). The NEBNext Ultra II RNA Library Prep Kit for Illumina (New England Biolabs, Ipswich, MA) was used for library preparation per manufacturer's protocol with the addition of NEBNext rRNA depletion option. Following fragmentation to 200-500 base pairs (bp) by Bioruptor Sonicator (Diagenode), reverse transcription (RT) was performed to convert RNA to cDNA, followed by end-repair, adaptor ligation, and PCR amplification of cDNA libraries per manufacturer's instruction (New England Biolabs, Ipswich, MA). Prior to sequencing, prepared libraries were assessed for fragment sizing on a Bioanalyzer using High Sensitivity DNA chip and re-quantified with Qubit. Library sequencing was performed on an Illumina HiSeq 4000 NGS System (Illumina, San Diego, CA) to generate 50 bp reads with 300 million paired-end reads and 30x read depth.

Bioinformatic analysis of RNA sequencing: *Read alignment with STAR.* Raw reads were de-multiplexed into FASTQ format using bcl2fastq software (Illumina) The FASTQ files were quality checked for called bases of each read. Pre-processing of raw data was performed by Trimmomatic option in Chipster where reads of quality below 20 were

eliminated. The Illumina adapters were removed from the 3' ends by *cutadapt* command-line program that operated under two adapter trimming algorithms (12). Trimmed reads were aligned to the *Mus musculus* genome mm10 using STAR RNA-seq aligner (version STAR_2.5.1 (13)). Of note, pre-processing of the reads was performed in conjunction with the mm10 mouse gene annotation file obtained from Ensembl (http://useast.ensembl.org/index. html). Reads with read lengths <35 were discarded. The read counts for individual genes were generated by *htseq-count* script derived from the HTseq framework v.0.6.1p1 that counts and calculates the read overlaps of genes within the mouse genome (14).

Identification of differentially-expressed genes (DEGs) by DESeq2. To identify DEGs, normalization for sequencing depth followed by variance-stabilizing transformation as well as differential expression calculations were performed via DESeq2 R Bioconductor package 1.16.1, which employed the Wald test to shrink Log-Fold-Changes (LFC) and their standard errors (15). The cut-off for determining significantly differentially expressed genes was determined with a stringency level where an FDR-adjusted $p<0.05$ and $|\log_2 FC| <1$ using the Benjamini-Hochberg method. In addition, genes with normalized counts < 100 were removed and not considered for future analysis. To execute principal-component analysis (PCA) and heatmap visualization, read counts were transformed to $\log_2$(count per million) and corrected for batch effects using limma package (16). In these analyses, PCA plots were made using the *pca()* function and hierarchical clustering with the *dist()* function.

Gene Set Enrichment Analysis (GSEA) & Gene Ontology (GO) Enrichment Analysis. To identify the enriched gene sets, GSEA was performed using the HTSAnalyzeR package

with a p-value cutoff of 0.05 after Benjamini-Hochberg correction (17). To identify overrepresented pathways operating within each sample, pathway analysis was performed using Metascape with Clarivate Analytics [http://metascape.org] with DEGs identified by DESeq2 as inputs (18). Outputs identified functionally related genes within characterized GO terms as well as coordinated changes in pathways significantly overrepresented within the datasets. In pathway network diagram, the node sizes represent the frequency of genes appearing in the indicated GO terms, whereas network edges indicate the inter-correlations and the overlaps between connected pathways.

Immune and Angiogenic Signatures. Several sets of (i) genes involved in G-MDSC phenotype (19), PMN granularity and surface markers (20), transcription factors (21), (ii) genes that were highly expressed in blood and lymphatic vessels (*angiotropic*) (22), and (iii) genes directly involved in vessel sprouting (*angiogenic*) (22) were manually curated from previously published datasets. To define the pathway-gene relationship within each biological sample, specific scores for each GO term involved in regulation of vessel development pathways and endothelial cell functions were computed via the number and the percentage of DEGs present/enriched in the specified GO terms.

Data availability. All original files for RNA-Seq data were submitted to NCBI's Gene Expression Omnibus database with the accession number GSE232217.

Whole-mount spinning-disk confocal microscopy: *Tissue preparation.* At the indicated experimental endpoints, CRC-bearing colons were exteriorized, dissected longitudinally to expose the epithelial lining of the lumen, washed several times to remove fecal matters and mucus, and secured using Vetbond tissue adhesive onto a silicon pedestal for imaging (3M, to minimize drifting movement). For imaging duration, tissue

was submerged into 37°C-warmed phenol red-free DMEM. All whole-mount imaging experiments were performed by spinning-disk confocal microscopy. To visualize healthy or tumor blood vessels, non-blocking anti-PECAM-1 Ab (CBL1337, EMD Millipore) conjugated to DyLight®-650 NHS Ester (62265, Thermo Fisher, Waltham, MA) was administered i.v. (retro-orbital) 30 min prior to imaging. To visualize the tumor cell interface, exteriorized whole-mount tumors were stained *in situ* with EpCAM antibody (clone G8.8, 118205, BioLegend) conjugated with PE or FITC.

Spinning-disk confocal imaging. The UltraVIEW VoX imaging system was built on an Olympus BX-51WI Fixed Stage illuminator and equipped with a Yokogawa CSU-X1-A1 spinning disk, a Hamamatsu EMCCD C9100-50 camera and a Modular Laser System with solid state diode lasers with DPPS modules for 488, 561, and 640 nm and the appropriate filters (all assembled by Perkin Elmer, Naperville, IL). Synchronization was managed by a Prosync 2 Syncronization Controller. Z-axis movement and objective positioning was controlled by Piezoelectric MIPOS100 System (Piezoystem Jena, Germany). Images were collected using a 20× water-immersion objective (1.00 numerical aperture). Volocity® software (Perkin Elmer) was used to drive the microscopy and acquire images, which were then analyzed using ImageJ. $Lyz2^{EGFP}$ is a well-accepted model for studying PMN trafficking where morphology can specifically identify PMNs. Of note, tissue $EGFP^{hi}$ tissue PMNs could be clearly distinguished from $EGFP^{low}$ tissue macrophages (by both morphology and GFP intensity), such that monocyte/macrophage signal did not interfere with tissue PMN analyses (**Supplemental Figure 1**). Data analysis was performed using ImageJ and Imaris software.

Endothelial tube formation assay: Tube formation assays were performed in triplicates in 96-well flat-bottom plates coated with 50μL of Matrigel (Cat:354234, Corning), where the matrigel was allowed to solidify for 20-30 minutes at 37°C prior to seeding. Immortalized murine (bENDs) and human endothelial cells (HUVECs) were cultured in DMEM (10% FBS) and Endothelial Cell Basal Medium (Cat:210-500, Sigma Alrich), respectively. In these assays, endothelial cells were seeded at low density of $1.5x10^4$ cells/well alone or in the presence of supernatant derived from FACS-sorted PMNs ($3x10^4$ cells) or human/murine recombinant proteins (MT1-MMP, 1-15μg/mL; OPN, 1-10μg/mL; VEGF, 100-1000ng/mL) with/without the addition of blocking monoclonal antibodies (anti-MT1/MMP mAb, 10μg/mL or anti-OPN mAb, 3μg/mL) or small-molecule inhibitors (GM6001, 10μM; NSC405020, 10μM). In these experiments, VEGF-supplemented and serum-free basal media were used as positive and baseline control, respectively. Formation of two-dimensional tubal and branching structures through a period of 5-6 hours (HUVEC) or 6-7 hours (bENDs) was monitored at intervals via bright-field microscope. For imaging, cells were gently washed with PBS and fixed with 1% PFA. Image analysis was carried out in ImageJ using a customized input “Angiogenesis Analyzer” (23).

Endothelial cell migration assay: Migration assays were performed in 24-well Transwell plates (Corning Costar, Cambridge, MA) with polycarbonate 6.5-mm membrane inserts of 8-μm pore size. The insert was pre-coated with a 75μg mixture of human plasma fibronectin (Cat:33016-015, Gibco Life Technologies) and collagen I (Cat:A1048301, Thermo Fisher) for 10-12 hours to mimic the highly dense stroma matrix in the tumor microenvironment. Prior to seeding of murine bENDs or human HUVECs, the insert

coating was re-hydrated for an hour in serum-free DMEM or Endothelial Cell Basal Medium (Sigma Aldrich, St. Louis, MO). To create chemoattractant gradients, supernatants derived from $5x10^4$ FACS-sorted PMNs/TANs or recombinant proteins were added to the bottom chambers with/without the addition of anti-OPN or anti-MMP14 neutralizing Abs. Subsequently, bENDs or HUVECs ($5x10^4$) were added to the upper chambers in serum-free media and allowed to migrate for 18-20 hours. At the endpoint, non-migrated cells on the top chambers were removed by cotton swab, and the inserts were gently washed with PBS, PFA-fixed (4%, 10 min), and permeabilized by methanol (20 min). Fixed migrated cells were stained with 0.5% Crystal Violet solution for 30 min and imaged by a bright-field microscope. The number of migrated endothelial cells was analyzed by ImageJ software. Data were shown as three independent experiments performed in duplicates for PMN-derived supernatants and in triplicates for media conditioned with recombinant proteins.

Bone marrow extraction and gradient-based neutrophil purification: Murine bone marrow cells were isolated as previously described (10, 24). Briefly, Bone marrow cells were flushed from the femur and tibia with serum-free Dulbecco modified Eagle's medium, and red blood cells were lysed by sequential treatment with 0.02% and 1.6% NaCl. Mouse BM-derived PMNs were further isolated and enriched to approximately 85% to 90% purity using Histopaque gradients (1077 and 1119; Sigma-Aldrich) as previously described (10).

***Ex vivo* co-culture of bone-marrow-derived PMNs with CRC or adjacent stroma:** Bone-marrow-derived PMNs, CRC tumors, and tumor-adjacent colon tissues were extracted from the same mice at early (week 7-8) or advanced CRC (week 13-14) time

points. Extraction procedures of BM PMNs and cancer cells were performed as described above, whereas extraction of colon crypts and lamina propria cells were described elsewhere (10, 24, 25). The experimental setup was conducted in 24-well plates with polycarbonate 6.5-mm membrane inserts of 0.4-μm pore size where cultured PMNs were physically separated from cancer cells or non-cancerous colon tissues. Single-cell isolates from cancer or non-cancerous colon tissues were seeded with 90-95% confluency ($1x10^6$) in bottom chambers, and BM-derived PMNs ($0.5x10^6$) from the same mouse were added to the top chambers at the ratio of 2 cancer cells (or adjacent colon cells) per 1 PMN cell. Following 24 hours of co-incubation, PMNs contained in the inserts were lysed by RNA Lysis Buffer (350μL) and RNA was extracted by RNeasy Mini Plus Kit (Qiagen). Quantitative RT-PCT was performed for genes encoding pro- and anti-angiogenic factors as identified by the RNA-Seq.

Cytospins and immunofluorescence of blood and tumor-associated neutrophils: Peripheral blood PMNs or TANs were enriched by FACS using $EpCAM^{neg}$ $CD45^+$ $CD11b^+$ $Lyz2^{EGFP}$ $Ly6G^{hi/int}$ gating, fixed with 4% paraformaldehyde followed by permeabilization using 1% Triton X-100, and stained with the relevant primary antibodies Osteopontin/OPN or MT1-MMP (10μg/ml, overnight, 4°C) followed by secondary antibodies (2hr, RT) and Hoechst, and concentrated onto non-charged slides by CytoSpin for mounting with FluorSave (EMD Millipore) prior to imaging. Imaging was performed using a Nikon (Tokyo, Japan) A1R confocal laser microscope equipped with a ×60 and ×100 Plan-Apochromat objective with a NA of 1.4 with a 0.25μm z-step (10 steps per field).

Data mining of CRC patient cohort: Gene expression CRC patient datasets published in The Cancer Genome Atlas (TCGA) were accessed through cBioPortal (26) for Cancer

Genomics (https://www.cbioportal.org/). RNA-sequencing data from a cohort of patients with CRC (n = 594 patients, TCGA PanCancer Atlas) were accessed using keywords "Bowel" and "Colorectal Adenocarcinoma (TCGA, PanCancer Atlas)." Transcript levels of vessel markers (*PECAM1, CDH5, VWF)* and PMN/Myeloid/TAN markers (*Itgam*, *S100A9, S100A8, CD14)* were constrained with a *z*-scores of 1.5 relative to all samples (log RNA-Seq V2 RSEM) to determine the correlation between vessel and neutrophil markers. Using similar constraints, transcript expression levels of TAN-enriched MMP (*MMP14*, *SPP1*) as well as novel TAN surface markers (*CD86*, *CD14*, *CD74*, CD33) and transcription factors (*SPI1*, *RUNX1*), were co-assessed with *PECAM1*, *VWF*, *CDH5*, *NOTCH3* transcript to determine correlations between TAN-specific factors and vessel marker in the same patient cohort. Patients with oncoprint of $SPP1^{high}$/$MMP14^{high}$ (z-score >1.5 compared to normal samples) was selected as altered group and compared with unaltered control group, in terms of tumor subtype, stages, and expression of PMN markers (*ITGAM*, *S100A8*, *S100A9*, *CD14*).

Statistics: Unless stated otherwise, all graphs were presented as mean ± S.E.M. Statistical differences for two groups comparisons were determined by two-tailed unpaired student's t test for normally distributed data and Mann-Whitney U test for non-parametric data. For three or more groups one-way ANOVA followed by Dunnett's or Tukey's multiple comparison was used. DEGs in bulk RNA-Seq experiments were analyzed by two-sided t test with Bayes moderation (eBayes function in R/bioconductor limma package (16)). Statistical significance was corrected for multiple testing by Benjamini-Hochberg method. Correlative gene expression analyses of the CRC Pan-Can patient cohort were performed at the cBioPortal (26) website using Spearman's and

Pearson's correlation test. For *in vivo* inhibition studies, the power to detect a significant difference among at least two groups was set at 80% (P = 1- β) with two-sided type I error α level of 0.05, with a calculated sample size of 6-8 mice per group at an effect size of 1.5. Power calculation and sample-size determination are performed in STATA and G*Power. All statistical analyses were performed using GraphPad Prism v.9.0.

Supplemental Figures:

Figure S1

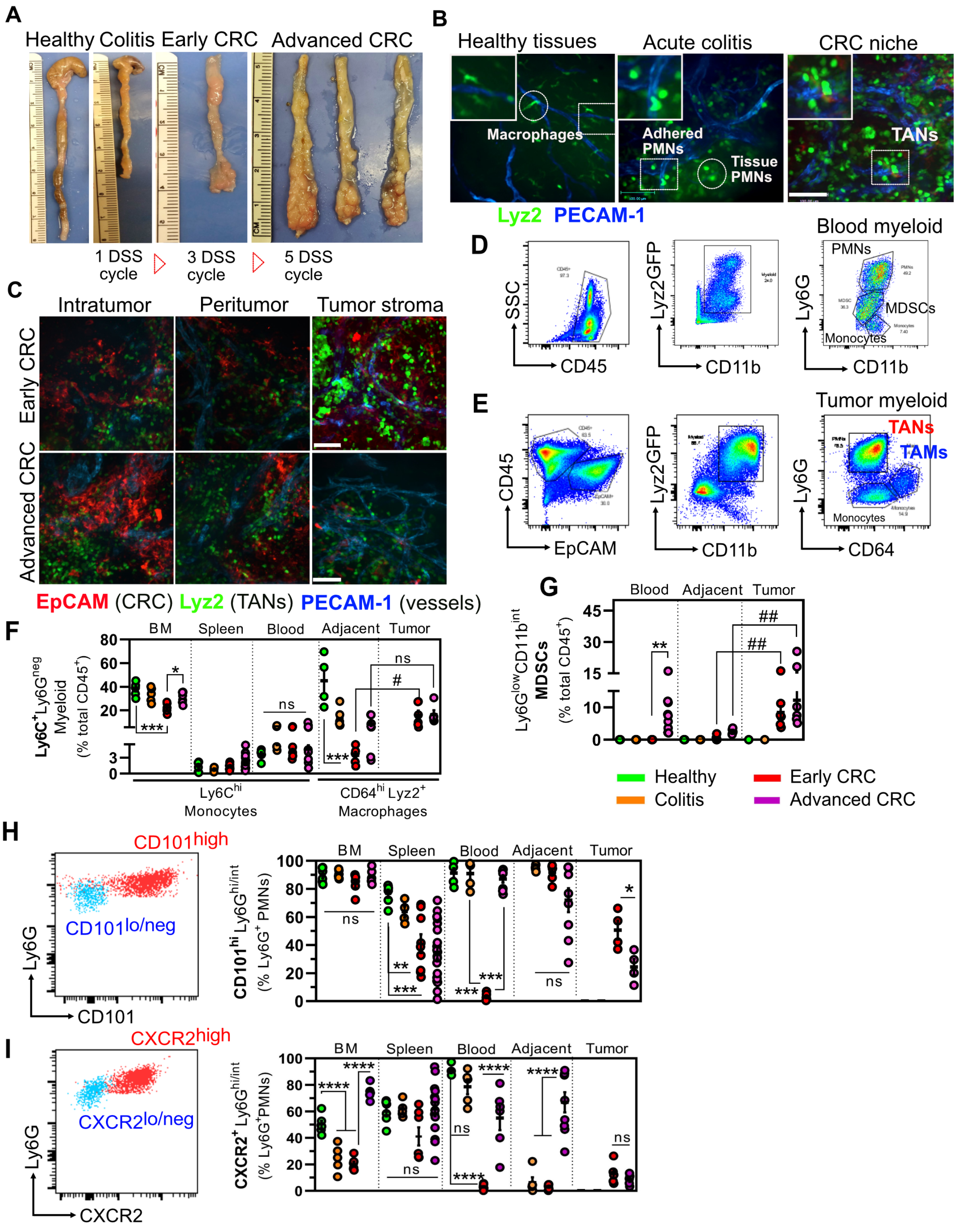

Supplemental Figure 1: Pathologic tissue accumulation and distinct phenotypes of peripheral blood PMNs and TANs during AOM/DSS-induced CRC development. (A) Macroscopic images of healthy, DSS-induced colitis (1 week), and AOM/DSS-induced early (8-10 weeks)/ advanced (15 weeks) tumor-bearing colons. **(B)** Whole-mount confocal imaging of AOM/DSS-induced CRC tumors in Lyz2^{EGFP} reporter mice show healthy tissue elongated/ramified macrophage morphology (*left*) and rounder PMNs infiltrating colitis-inflamed tissues (*middle*) and more so the CRC niche (*right*). Insets are high-power images of tissue macrophages (*left*) and PMNs interacting with inflamed (*middle*) or tumor vasculature (*right*). Scale bar 100μm. **(C)** Whole-mount confocal imaging of AOM/DSS-induced CRC tumors in Lyz2^{EGFP} reporter mice show increased TAN (green) accumulation in advanced compared to early tumors in intratumoral, peripheral, and tumor adjacent stromal regions. ECs were visualized by PECAM-1 (blue) and tumor cells by EpCAM (red) staining on the luminal side of the colon. Scale bar, 100μm. **(D)** Gating strategy of circulating myeloid cells (PMNs, PMN-MDSCs, monocytes) and **(E)** intratumoral myeloid cells (TANs, TAMs) in AOM/DSS-induced advanced CRC. (**F**) Quantification of Ly6C^{hi}/Ly6G^{neg} monocytes in BM, blood, and spleen and CD64^{hi}/ Lyz2^{int} macrophages in tumors and adjacent tissues during colitis to CRC transition. (**G**) Quantification of PMN-MDSCs (Ly6G^{lo}/Lyz2^{lo}/CD11b^{int}) in peripheral blood, tumor, and adjacent tissues during CRC progression. **(H)** Quantification of CD101^{hi}/Ly6G$^{hi/int}$ and **(I)** CXCR2^{+}/Ly6G$^{hi/int}$ PMNs across specified tissue compartments during CRC transition. Representative overlay diagrams of CD101^{hi} versus CD101^{lo} or CXCR2^{hi} versus CXCR2^{neg} are shown. For all flow analyses n=5 healthy, n=6 colitis and n=8-12 early/advanced CRC bearing mice. * $p<0.05$, ** $p<0.01$, *** $p<0.001$, significance between

disease stages; ## p<0.01, significance between cancer and adjacent tissues. ns non-significant (One-way ANOVA with Tukey's multiple comparison test). Data are presented as mean ± SEM.

Figure S2

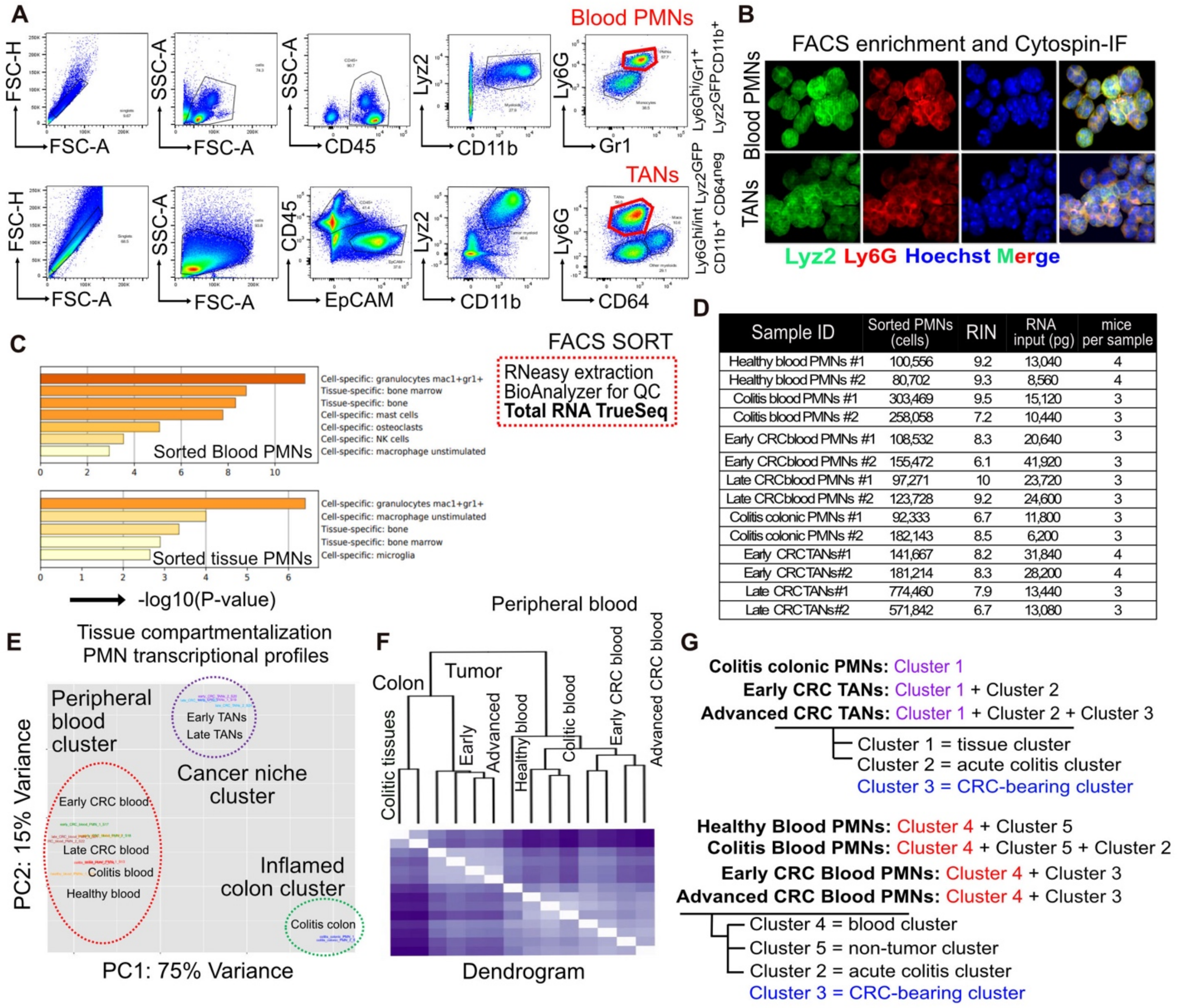

Sample ID	Sorted PMNs (cells)	RIN	RNA input (pg)	mice per sample
Healthy blood PMNs #1	100,556	9.2	13,040	4
Healthy blood PMNs #2	80,702	9.3	8,560	4
Colitis blood PMNs #1	303,469	9.5	15,120	3
Colitis blood PMNs #2	258,058	7.2	10,440	3
Early CRCblood PMNs #1	108,532	8.3	20,640	3
Early CRCblood PMNs #2	155,472	6.1	41,920	3
Late CRCblood PMNs #1	97,271	10	23,720	3
Late CRCblood PMNs #2	123,728	9.2	24,600	3
Colitis colonic PMNs #1	92,333	6.7	11,800	3
Colitis colonic PMNs #2	182,143	8.5	6,200	3
Early CRCTANs#1	141,667	8.2	31,840	4
Early CRCTANs#2	181,214	8.3	28,200	4
Late CRCTANs#1	774,460	7.9	13,440	3
Late CRCTANs#2	571,842	6.7	13,080	3

Supplemental Figure 2: Gating strategy and FACS approach for transcriptomic analysis of peripheral blood PMNs and TANs. (A) FACS gating approach for enrichment of peripheral blood PMNs (*top*) and CRC TANs (*bottom*) using Lyz2^{EGFP} myeloid reporter mice. Peripheral blood PMNs were gated from CD45^{+} immune cells as Ly6G$^{hi/int}$/Gr1^{+}/Lyz2^{EGFP}/CD11b^{+} and TANs as Ly6G$^{hi/int}$/Lyz2^{EGFP}/CD11b^{+}/CD64^{neg} (outlined in red). Due to limited PMN RNA quantity, PMNs/TANs were pooled from n=3-4 mice for each RNA-Seq replicate to ensure sufficient RNA extraction and construction of the cDNA library. **(B)** High-power representative confocal microscopy images of FACS-

sorted control PMNs showing expected hypersegmented and/or banded nuclear morphology (stained with Hoechst) confirmed PMN gating strategy (n=4 independent experiments). **(C)** Enrichment of cell type-specific gene sets of FACS-sorted cells confirmed granulocytes (Mac1$^+$/Gr1$^+$) as top significance score for sorted peripheral blood PMNs ($p<10^{-10}$) and TANs ($p<10^{-6}$). **(D)** Specimen IDs and description of each RNA-Seq experiment performed for all seven PMN conditions (in duplicates). **(E)** Two-dimensional PCA of DEG variance in all RNA-Seq specimens segregates PMN transcriptional profiles into 3 compartments corresponding to peripheral blood, colon tissue and the CRC niche. **(F)** Correlation matrix shown as dendrogram and **(G)** annotation of 14 RNA-Seq specimens. Unbiased clustering separated PMNs based on their localization to peripheral blood and colon/CRC tissue, displaying corresponding gene cluster modules indicated in Figure 1G-H.

Figure S3

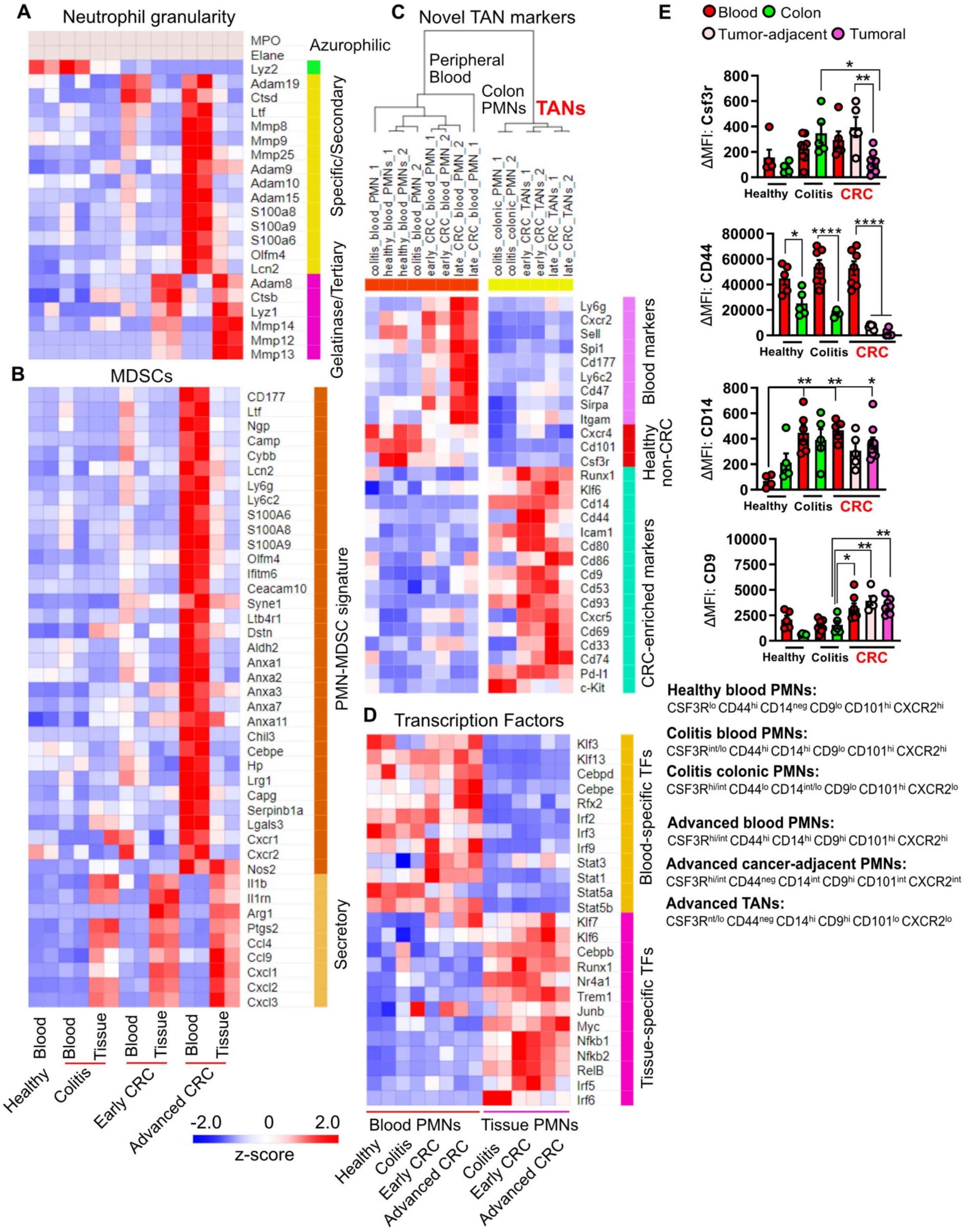

A
Neutrophil granularity
MPO
Elane
Azurophilic
Lyz2
Adam19
Ctsd
Ltf
Mmp8
Mmp9
Mmp25
Adam9
Adam10
Adam15
S100a8
S100a9
S100a6
Olfm4
Lcn2
Adam8
Ctsb
Lyz1
Mmp14
Mmp12
Mmp13
Specific/Secondary
Gelatinase/Tertiary
B
MDSCs
CD177
Ltf
Ngp
Camp
Cybb
Lcn2
Ly6g
Ly6c2
S100A6
S100A8
S100A9
Olfm4
Ifitm6
Ceacam10
Syne1
Ltb4r1
Dstn
Aldh2
Anxa1
Anxa2
Anxa3
Anxa7
Anxa11
Chil3
Cebpe
Hp
Lrg1
Capg
Serpinb1a
Lgals3
Cxcr1
Cxcr2
Nos2
Il1b
Il1rn
Arg1
Ptgs2
Ccl4
Ccl9
Cxcl1
Cxcl2
Cxcl3
PMN-MDSC signature
Secretory
Blood
Blood
Tissue
Blood
Tissue
Blood
Tissue
Healthy
Colitis
Early CRC
Advanced CRC
-2.0
0
2.0
z-score
C
Novel TAN markers
Peripheral Blood
Colon PMNs
TANs
colitis_blood_PMN_1
healthy_blood_PMNs_1
healthy_blood_PMNs_2
colitis_blood_PMN_2
early_CRC_blood_PMN_1
early_CRC_blood_PMN_2
late_CRC_blood_PMN_2
late_CRC_blood_PMN_1
colitis_colonic_PMN_1
colitis_colonic_PMN_2
early_CRC_TANs_1
early_CRC_TANs_2
late_CRC_TANs_1
late_CRC_TANs_2
Ly6g
Cxcr2
Sell
Spi1
Cd177
Ly6c2
Cd47
Sirpa
Itgam
Cxcr4
Cd101
Csf3r
Runx1
Klf6
Cd14
Cd44
Icam1
Cd80
Cd86
Cd9
Cd53
Cd93
Cxcr5
Cd69
Cd33
Cd74
Pd-l1
c-Kit
Blood markers
Healthy non-CRC
CRC-enriched markers
D
Transcription Factors
Klf3
Klf13
Cebpd
Cebpe
Rfx2
Irf2
Irf3
Irf9
Stat3
Stat1
Stat5a
Stat5b
Klf7
Klf6
Cebpb
Runx1
Nr4a1
Trem1
Junb
Myc
Nfkb1
Nfkb2
RelB
Irf5
Irf6
Blood-specific TFs
Tissue-specific TFs
Blood PMNs
Tissue PMNs
Healthy
Colitis
Early CRC
Advanced CRC
Colitis
Early CRC
Advanced CRC
E
Blood
Colon
Tumor-adjacent
Tumoral
ΔMFI: Csf3r
800
600
400
200
0
Healthy
Colitis
CRC
ΔMFI: CD44
80000
60000
40000
20000
0
Healthy
Colitis
CRC
ΔMFI: CD14
800
600
400
200
0
Healthy
Colitis
CRC
ΔMFI: CD9
10000
7500
5000
2500
0
Healthy
Colitis
CRC
Healthy blood PMNs:
CSF3R^{lo} CD44^{hi} CD14^{neg} CD9^{lo} CD101^{hi} CXCR2^{hi}
Colitis blood PMNs:
CSF3R$^{int/lo}$ CD44^{hi} CD14^{hi} CD9^{lo} CD101^{hi} CXCR2^{hi}
Colitis colonic PMNs:
CSF3R$^{hi/int}$ CD44^{lo} CD14$^{int/lo}$ CD9^{lo} CD101^{hi} CXCR2^{lo}
Advanced blood PMNs:
CSF3R$^{hi/int}$ CD44^{hi} CD14^{hi} CD9^{hi} CD101^{hi} CXCR2^{hi}
Advanced cancer-adjacent PMNs:
CSF3R$^{hi/int}$ CD44^{neg} CD14^{int} CD9^{hi} CD101^{int} CXCR2^{int}
Advanced TANs:
CSF3R$^{nt/lo}$ CD44^{neg} CD14^{hi} CD9^{hi} CD101^{lo} CXCR2^{lo}

Supplemental Figure 3: Distinct gene signatures of peripheral blood PMNs and TANs in tumor-bearing mice. (A) Row-scaled expression analyses with k-means clustering of PMN DEGs representing PMN granularity (19, 20), **(B)** MDSC signature (19), **(C)** novel surface/ intracellular markers (19), and **(D)** transcription factors (20, 21). Colors are scaled row-wise with a z-score from -2 to +2. **(E)** Immunophenotyping analyses of blood and tissue PMNs versus TANs by flow cytometry defined distinct protein signatures based on common PMNs (Csf3r/G-CSFR) and RNAseq-identified TAN markers (CD44, CD14, CD9) (n=4-7 mice for four independent experiments). Relative mean fluorescence intensity (MFI) compared to FMO control of the specified markers were shown. Six immunophenotypes of PMNs and TANs were listed by combining mature/immature markers (27) (CD101, CXCR2) with RNAseq-identified markers (C44, CD14, CD9, CSF3R). * $p<0.05$, ** $p<0.01$, *** $p<0.001$, **** $p<0.0001$, significance between disease stages and tissue conditions (one-way ANOVA with Tukey's multiple comparison test). Data are presented as mean ± SEM.

Figure S4

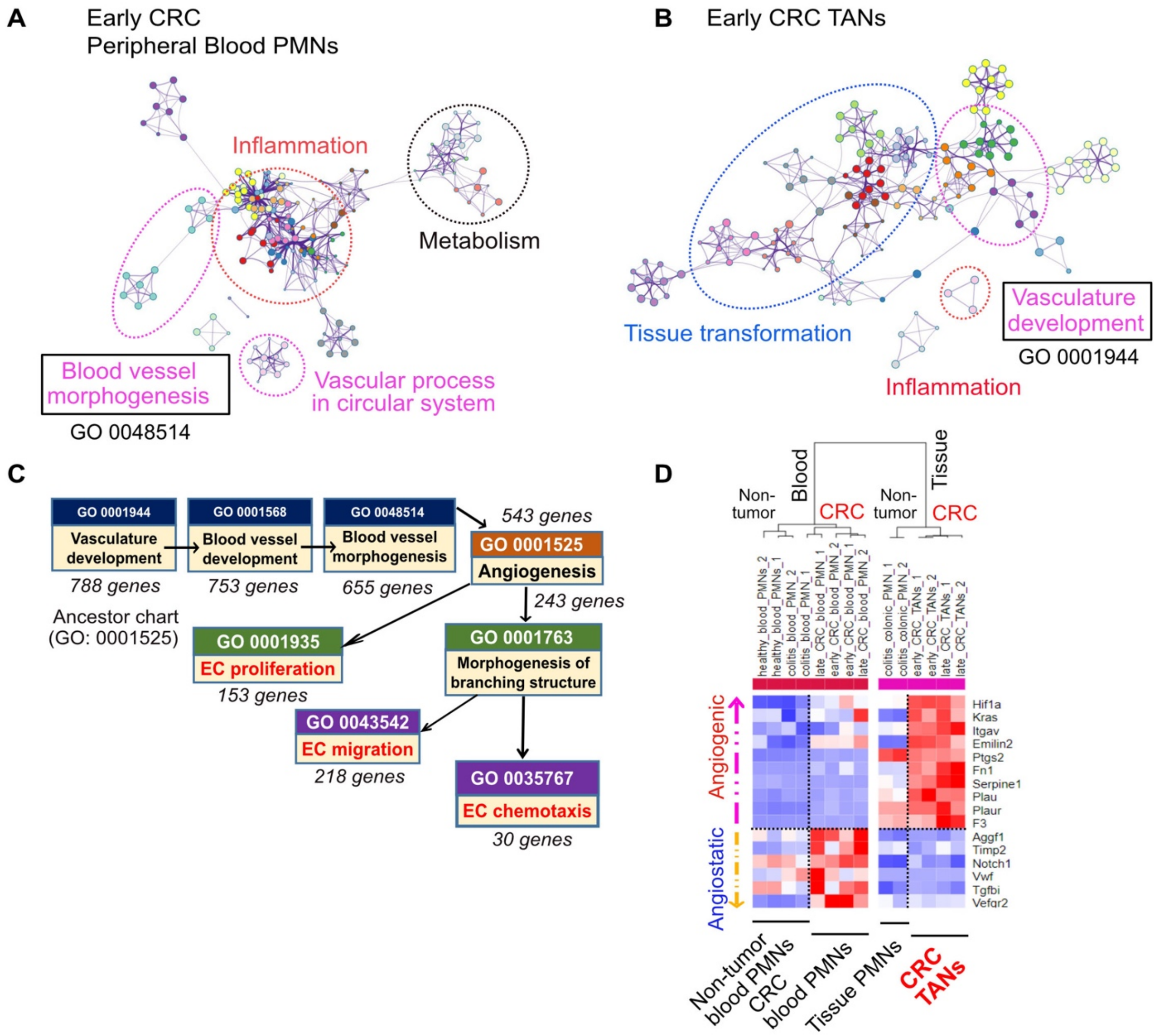

Supplemental Figure 4: Pathways and gene sets involved in hypoxic response and vasculature development are overrepresented in TANs. GO network analyses of enriched biological processes in **(A)** peripheral blood PMNs and **(B)** TANs present in early CRC. Node size reflects significance of the enrichment test, and the edges reflect overlap of GO terms involved in the interconnected biological processes. Clusters of major GO terms are outlined by dotted circles and annotated. **(C)** Ancestor GO chart of

Angiogenesis (GO:0001525) indicates hierarchy of upstream and downstream GO terms and the numbers of involved genes. **(D)** Row-scaled expression heatmap representation of DEGs associated angiogenic versus angiostatic intracellular factors (22). Colors are scaled row-wise with a z-score from -2 to +2.

Figure S5

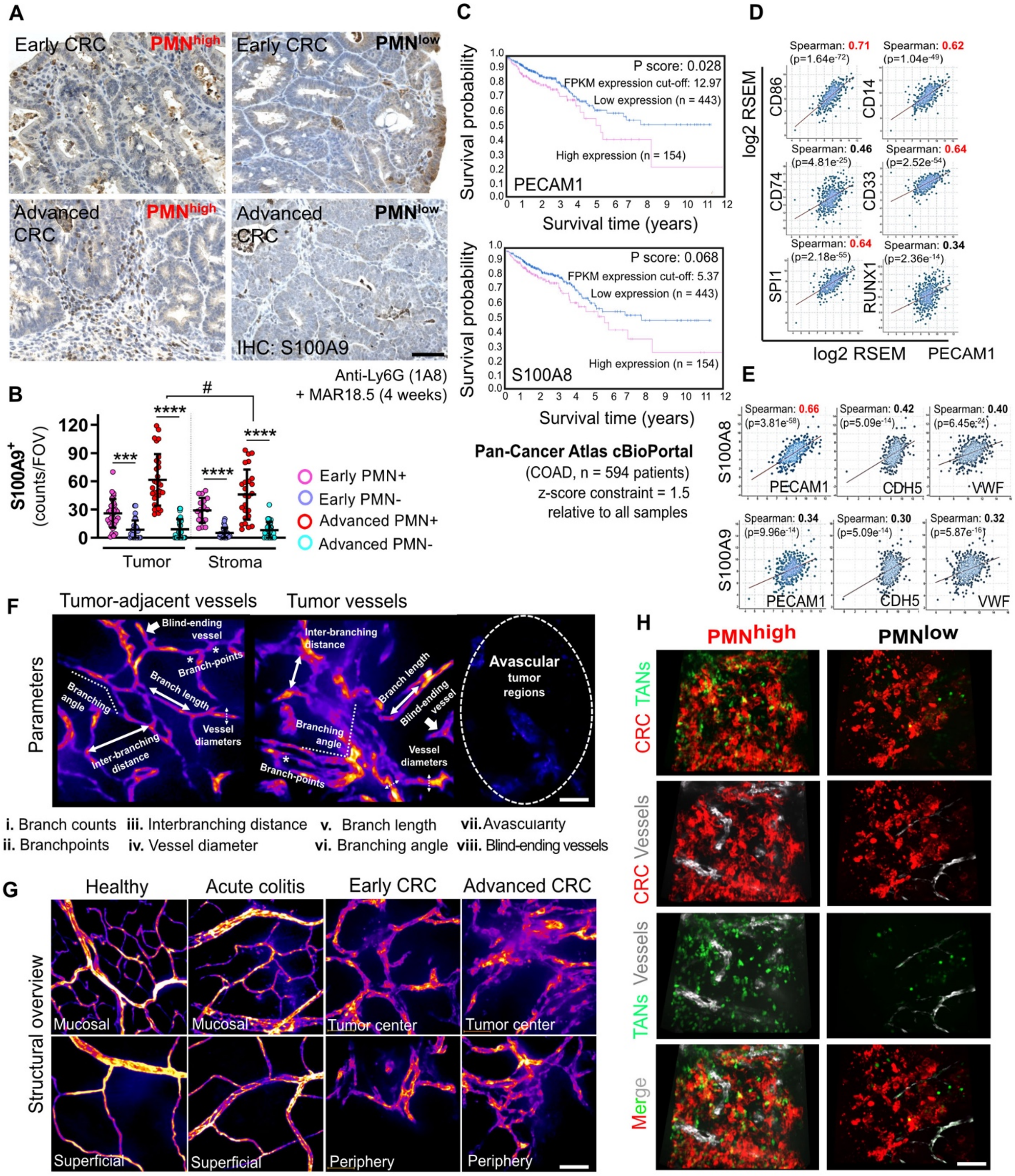

Supplemental Figure 5: Correlative analyses of TAN and vessel markers in murine AOM/DSS model and CRC patient cohort. (A) Representative IHC images of S100A9-

positive TANs in AOM/DSS-induced tumors treated with IgG isotype control (PMN^{high}) or anti-Ly6G/MAR18.5 (PMN^{low}). PMN depletion is evident in both early and advanced CRC. Scale bar 20μm. Images representative of 5-7 tumors per mouse for 6-8 mice in three independent experiments. **(B)** Quantification of $S100A9^{+}$ cells per FOV in intratumoral and stromal regions of PMN^{high} vs PMN^{low} tumors. n=40-50 FOVs for 5-6 tumors per tumor condition. *** p<0.001, **** p<0.0001, significance between PMN^{high} and PMN^{low}; # p<0.05, significance between intratumor and stromal area (one-way ANOVA with Tukey's multiple comparison test). **(C)** Kaplan-Meier survival analysis of TCGA cohort of Colorectal Adenocarcinoma (COAD) patients (26). Patients were stratified based on FPKM expression levels of PECAM-1 (cut-off = 12.97) or S100A8 (cut-off = 5.37). Patients with lower PECAM-1 or S100A8 (n = 443) had significantly better survival than those with higher expression (n = 154). **(D)** Correlative co-expression analyses of several RNAseq-identified TAN markers and the established vessel marker PECAM-1. **(E)** Correlative co-expression analyses of PMN markers S100A8 (*top*) and S100A9 (*bottom*) with three established vascular markers (PECAM-1, CDH5/VE-Cadherin and VWF). Gene expression (log_2RSEM) was analyzed using a cohort of 594 COAD patients obtained from the Pan-Cancer Atlas in cBioPortal (26). A z-score constraint of 1.5 relative to all samples was set for expression of each analyzed gene. Spearman's correlation co-efficient with corresponding p-value was determined for each gene and color-coded in *red* if was greater than 0.5. **(F)** Demarcation of the eight architectural parameters that are used to quantitatively assess vascular structures are shown in both tumor adjacent (*left*) and intratumoral regions (*middle*). Representative tumor avascular region is shown in the right panel. **(G)** Confocal images of PECAM-1-positive vessels in healthy versus inflamed

superficial and deep mucosa of the colon, as well as in early versus advance CRC tumors at center and periphery regions. **(H)** Representative 3D reconstruction of the CRC niche and tumor vasculature. Vessels and tumor cells were stained for PECAM-1 (white) and EpCAM (red) in Lyz2^{EGFP} reporter mice (PMNs, green). For all images, the scale bars are 50μm and images are representative of n=6-8 tumors. Data are presented as mean ± SEM.

Figure S6

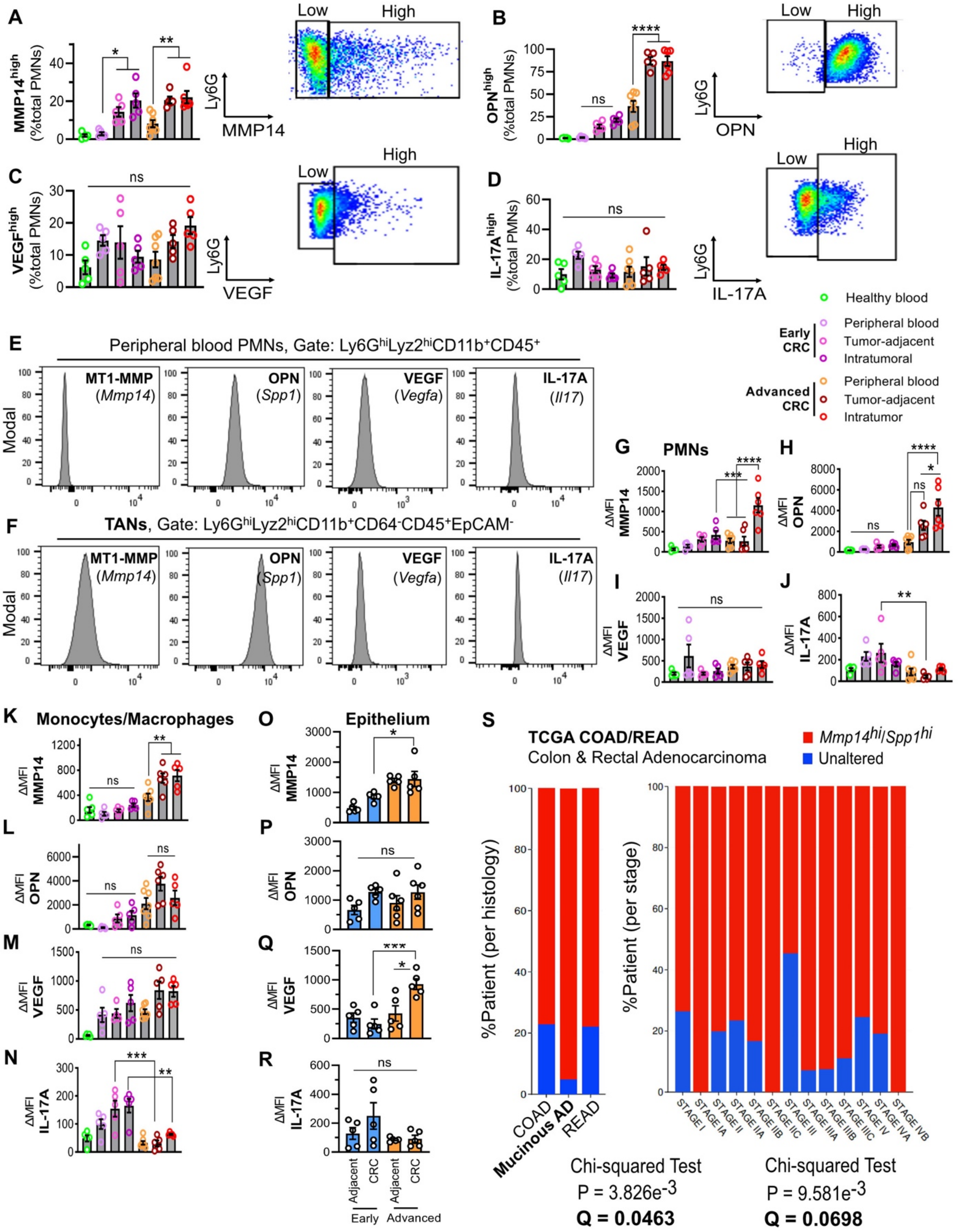

A
MMP14high (%total PMNs)
Low
High
Ly6G
MMP14
B
OPNhigh (%total PMNs)
Ly6G
OPN
C
VEGFhigh (%total PMNs)
Ly6G
VEGF
D
IL-17Ahigh (%total PMNs)
Ly6G
IL-17A
Healthy blood
Early CRC
Peripheral blood
Tumor-adjacent
Intratumoral
Advanced CRC
Peripheral blood
Tumor-adjacent
Intratumor
E
Peripheral blood PMNs, Gate: Ly6GhiLyz2hiCD11b+CD45+
MT1-MMP (Mmp14)
OPN (Spp1)
VEGF (Vegfa)
IL-17A (Il17)
Modal
F
TANs, Gate: Ly6GhiLyz2hiCD11b+CD64-CD45+EpCAM-
G
PMNs
ΔMFI MMP14
H
ΔMFI OPN
I
ΔMFI VEGF
J
ΔMFI IL-17A
K
Monocytes/Macrophages
L
M
N
O
Epithelium
P
Q
R
Adjacent
CRC
Early
Advanced
S
TCGA COAD/READ
Colon & Rectal Adenocarcinoma
Mmp14hi/Spp1hi
Unaltered
%Patient (per histology)
COAD
Mucinous AD
READ
%Patient (per stage)
STAGE I
STAGE IA
STAGE II
STAGE IIA
STAGE IIB
STAGE IIC
STAGE III
STAGE IIIA
STAGE IIIB
STAGE IIIC
STAGE IV
STAGE IVA
STAGE IVB
Chi-squared Test
P = 3.826e-3
Q = 0.0463
Chi-squared Test
P = 9.581e-3
Q = 0.0698

Supplemental Figure 6: Validation of MMP14 and OPN expression in blood PMNs and CRC TANs. (A-D) Flow cytometry analyses in healthy, colitis inflamed and CRC bearing Lyz2^{EGFP} reporter mice. Percentage of MMP14^{high}, OPN^{high}, VEGF^{high} and IL-17A^{high} PMNs/TANs (gated on Lyz2^{EGFP} Ly6G+) was quantified in the specified tissue compartments by intracellular staining and flow cytometry. Cell portions with high expressions were gated based on FMO control of tissues collected from the same conditions (shown in insets on the right). **(E-F)** Representative MFI histograms (Modal) of PMNs and TANs and **(G-J)** their respective quantification of MMP14, OPN, VEGF and IL-17 expression in each tissue compartments. **(K-N)** Expression of MMP14, OPN, VEGF and IL-17 was similarly assessed in by flow cytometry in monocytes/macrophages and **(O-R)** in intestinal epithelial/tumor cells. For all flow cytometry experiments, data are shown as ΔMFI relative to FMO controls. n =5 independent experiments. * $p<0.05$, ** $p<0.01$; **** $p<0.0001$, ns non-significant (Kruskal-Wallis test with Dunnet's multiple comparison test). **(S)** COAD patients from Pan-Cancer Atlas (cBioPortal) were analyzed for MMP14 and OPN expression and stratified into MMP14^{hi}/SPP1^{hi} (*red*, n=469) or unaltered group with baseline expression (*blue*, n=123). MMP14^{hi}/SPP1^{hi} patients were more likely to have aggressive/stroma-rich mucinous CRC sub-type (q = 0.0463) and trending to advanced CRC stages (Stage IIIA-C or Stage IVA-B, q = 0.0698, analyzed by Chi-Squared test and adjusted by patient numbers). Both histological features are known to associate with distant metastases and poor clinical outcomes. Data are presented as mean ± SEM.

Figure S7

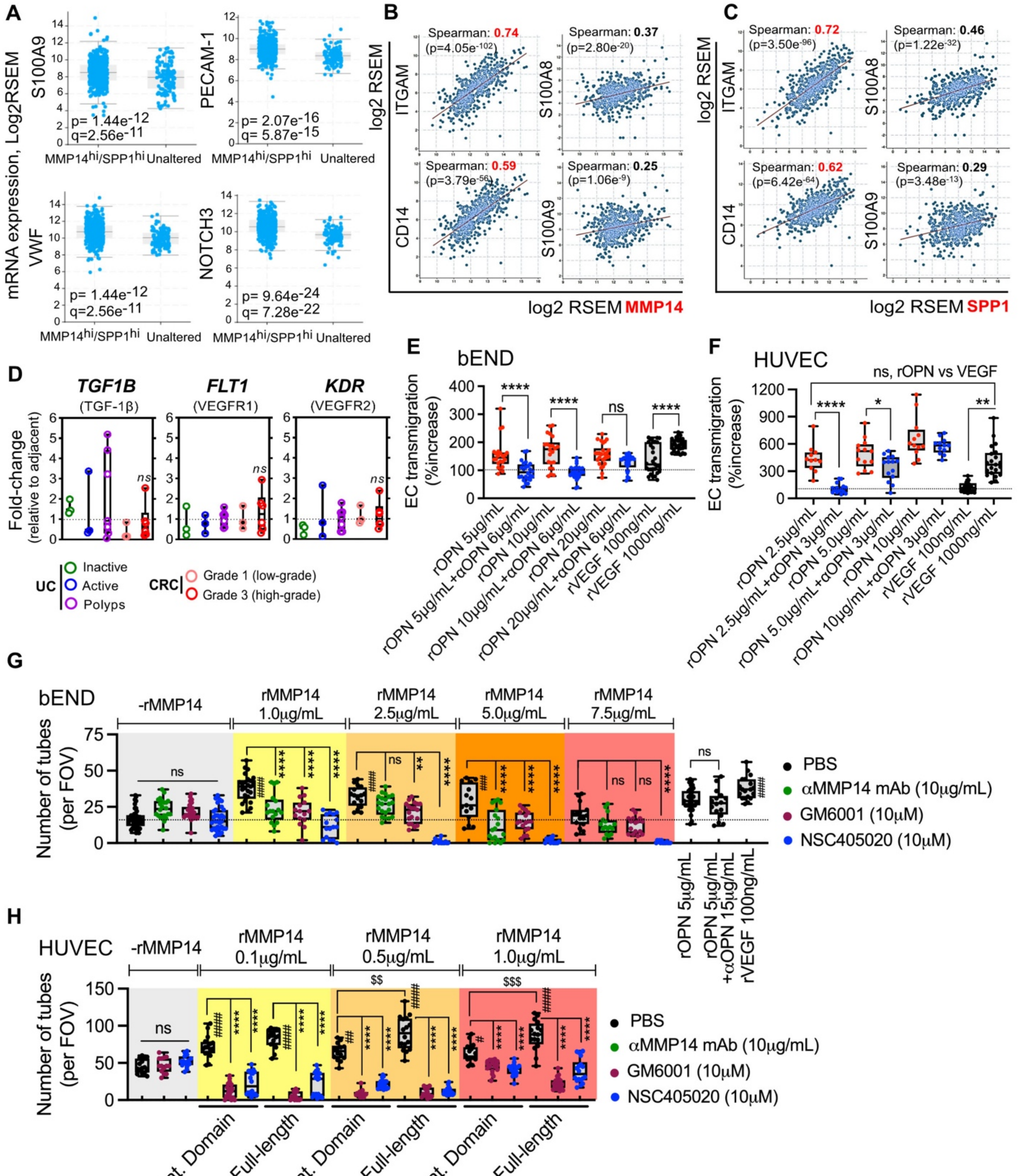

Supplemental Figure 7: MMP14/SPP1 expression and their non-overlapping angiogenic functions. (A) COAD patients from Pan-Cancer Atlas (cBioPortal (26)) with

elevated MMP14/SPP1 expression were correlated with significantly higher transcript levels of PMN (S100A9) and EC (PECAM-1, VWF, NOTCH3) genes, where p and adjusted q-values are shown per analysis. **(B)** mRNA expression analyses of several other key angiogenic factors including *TGFB1* and two VEGR receptors *FLT1*/VEGFR1 and *KDR*/VEGFR2 in biopsied tissue from non-active/active UC (n=3 and 10 patients) and low/high grade CRC (n=3 and 6 patients). **(C)** Co-expression analyses of MMP14 and **(D)** SPP1 with three established PMN/TAN markers (ITGAM, S100A8, S100A9) and one RNAseq-identified marker (CD14). Data shown as log2 RSEM with a z-score constraint of 1.5 for the specified genes across the COAD patient cohort (cBioPortal). Spearman's correlation co-efficient with corresponding p-value was determined for each parameter and color-coded in *red* if greater than 0.5. Expression was normalized to adjacent non-inflamed/non-cancerous tissues (two-sided student's t test, ns non-significant). **(E)** Whisker and box plots showing quantification of individual FOVs of bEND and **(F)** HUVEC EC migration relative to serum-free control condition (with recombinant VEGF as positive control, n=35-50 FOVs as technical duplicates for three independent experiments, one-way ANOVA with Tukey's multiple comparison test). * $p<0.05$, ** $p<0.01$; **** $p<0.0001$, ns non-significant, significance between blocking versus non-blocking conditions. **(G)** Quantification of bEND tube formation in response to increased concentrations of rMMP14 or rOPN with/without MMP14 inhibition by anti-MMP14 blocking mAb (10μg/mL), pan-MMP catalytic inhibitor GM6001 (10μM), or MMP14 allosteric inhibitor NSC405020 (10μM). **(H)** Quantification of HUVEC tube formation in the presence of catalytic domain (CD) or full-length (FL) rMMP14 with/without MMP14 inhibition by anti-MMP14 mAb, GM6001, or NSC405020. **** $p<0.0001$, ns non-

significant, significance between untreated versus respective inhibitory conditions; #, $p<0.05$, ### $p<0.001$, ####, $p<0.0001$; significance between rMMP14 treatment versus serum-free; $$ $p<0.01$, $$$ $p<0.001$, significance between the effects of catalytic domain versus full-length rMMP14 (one-way ANOVA with Tukey's multiple comparison test, n=25-40 FOVs for three independent experiments).

Figure S8

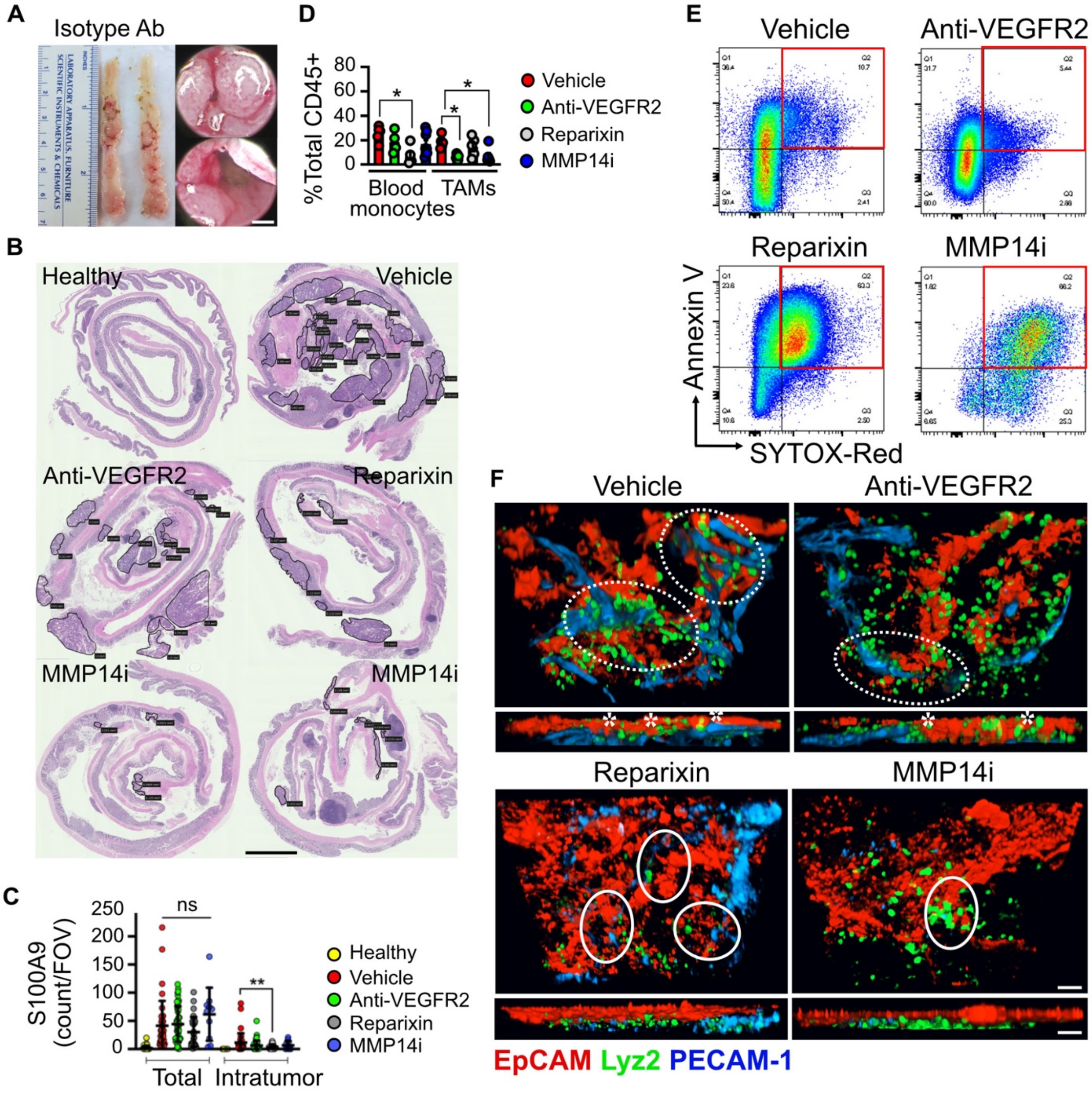

Supplemental Figure 8: The impact of pharmacological intervention on tumor immune cells and vasculature. (A) Representative macroscopic images of an excised colon and high-resolution endoscopic images of advanced CRC (week 15) treated with IgG-isotype control. Scale bar 1mm. Images representative of n=4 tumors per condition. **(B)** Representative H&E images of Swiss roll preparations of healthy or tumor-bearing

colons (advanced CRC, week 15, indued by AOM/DSS) with/without the specified drug treatment. Developing tumors are highlighted by the black dotted outlines. Scale bar 500μm. Images representative of n=4 tumors per condition. (**C)** IHC-based quantification of total (stroma and tumor) and intratumoral S100A9-positive PMNs with the specified drug treatments. * $p<0.05$, ** $p<0.01$, *** $p<0.001$, **** $p<0.0001$, significance between specified treatment and vehicle/isotype control (Kruskal-Wallis's test with Dunnett's multiple comparison test). **(D)** Quantification of peripheral blood monocytes ($Ly6C^{hi}$/ $Ly6G^{neg}$/$CD11b^{+}$) and TAMs (LyG^{neg}/$CD64^{hi}$) in response to specified treatments at tumor endpoints. * $p<0.05$, significance between specified treatment and vehicle/isotype control (one-way ANOVA with Tukey's multiple comparison test). Data are presented as mean ± SEM. **(E)** Representative flow cytometry micrographs showing gating strategy for apoptotic/dead cell quantification using Annexin V and SYTOX Red (red boxes outline quadrants of late apoptotic/dead cells, quantification shown in Figure 6G). Images representative of n=4 independent experiments. **(F)** 3D reconstruction (rendering planes in XZ direction) and Z-stack projections of whole-mount tumor tissues. Images are serially acquired using a spinning-disk confocal microscopy in advanced AOM/DSS-induced CRC with the specified treatment. Dotted circles highlight contact regions of TAN ($Lyz2^{EGFP}$, *green*), tumor vessels, (PECAM-1, *blue*) and tumor cells (EpCAM, *red*) in vascularized tumors. Solid circles show reduced vessel penetrations or presence in Reparixin- and MMP14i-treated tumors. Asterisks in XZ orthogonal views (*below*) mark TAN-vessel-tumor cell contacts in vehicle and anti-VEGFR2 groups, which are lost with Reparixin and MMP14 inhibition. For each image, 100μm stacks were generated using 1μm-focal depth steps. Images representative of three independent experiments.

Figure S9

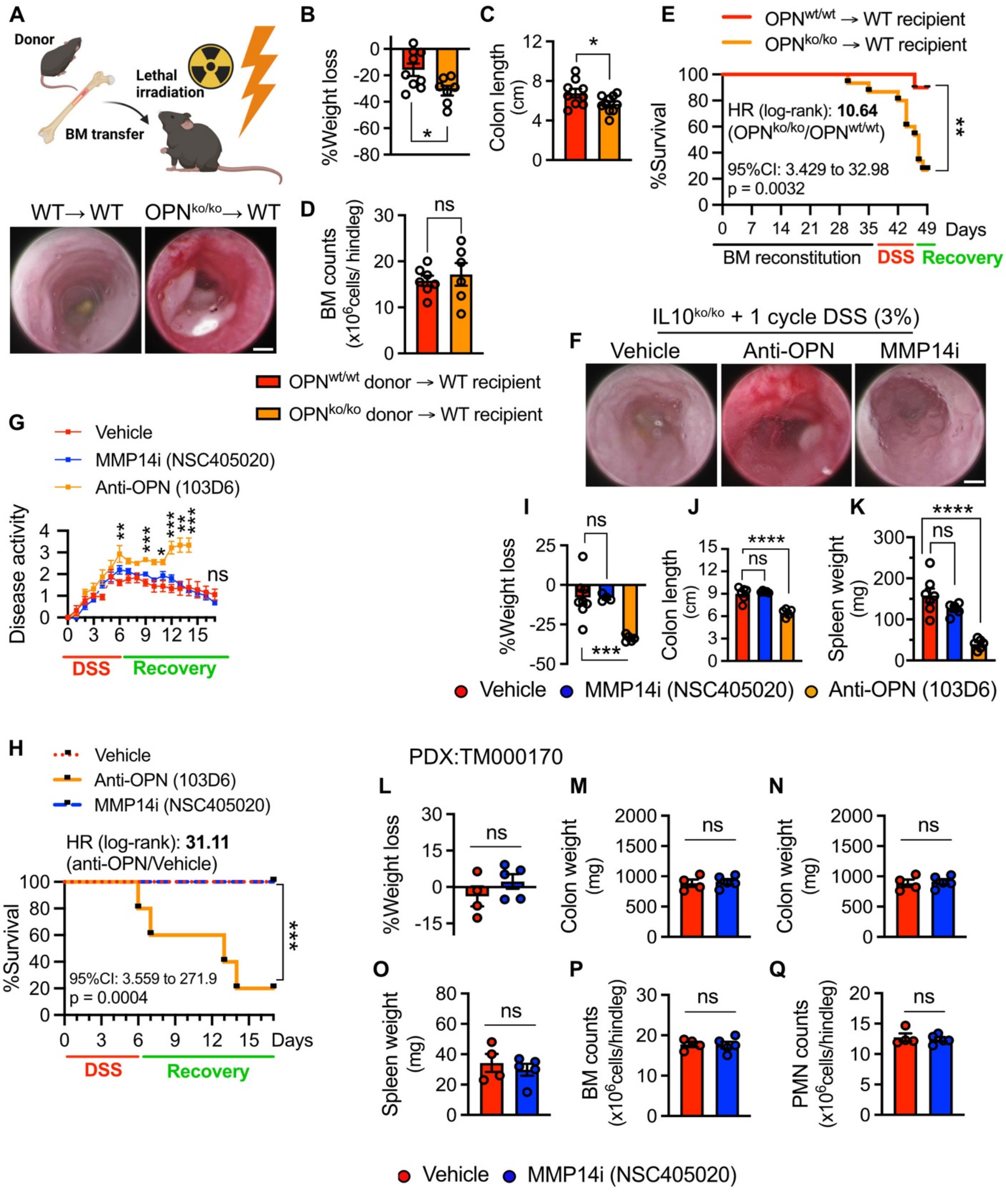

A
Donor
Lethal irradiation
BM transfer
WT→ WT
OPNko/ko→ WT
B
%Weight loss
C
Colon length (cm)
D
BM counts (x10^6cells/ hindleg)
ns
OPNwt/wt donor → WT recipient
OPNko/ko donor → WT recipient
E
OPNwt/wt → WT recipient
OPNko/ko → WT recipient
%Survival
HR (log-rank): 10.64
(OPNko/ko/OPNwt/wt)
95%CI: 3.429 to 32.98
p = 0.0032
Days
BM reconstitution
DSS
Recovery
IL10ko/ko + 1 cycle DSS (3%)
F
Vehicle
Anti-OPN
MMP14i
G
Vehicle
MMP14i (NSC405020)
Anti-OPN (103D6)
Disease activity
DSS
Recovery
I
%Weight loss
J
Colon length (cm)
K
Spleen weight (mg)
Vehicle
MMP14i (NSC405020)
Anti-OPN (103D6)
H
Vehicle
Anti-OPN (103D6)
MMP14i (NSC405020)
HR (log-rank): 31.11
(anti-OPN/Vehicle)
%Survival
95%CI: 3.559 to 271.9
p = 0.0004
Days
DSS
Recovery
PDX:TM000170
L
%Weight loss
M
Colon weight (mg)
N
Colon weight (mg)
O
Spleen weight (mg)
P
BM counts (x10^6cells/hindleg)
Q
PMN counts (x10^6cells/hindleg)
Vehicle
MMP14i (NSC405020)

Supplemental Figure 9: OPN is essential for disease resolution and survival following DSS-induced colitis. (A) Schematic of bone-marrow (BM) transfer experiments (*top*). $OPN^{ko/ko}$ or $OPN^{wt/wt}$ immune cells were transferred into irradiated WT recipients. Following BM reconstitution animals were treated with AOM/DSS. Representative colonoscopy images of $OPN^{ko/ko}$ and wildtype BM chimeras following one 3% DSS cycle (*bottom*). (**B**) Quantification of percent weight loss, **(C)** colon length, **(D)** absolute BM cell counts after one cycle of DSS (two-sided student's t-test, * $p<0.05$, ns non-significant, significance between $OPN^{ko/ko}$ versus wildtype chimeras). **(E)** Kaplan-Meier plot comparing survival differences of $OPN^{ko/ko}$ versus wildtype chimera during a 49-day period following BM transfer. A log-rank (Mantel-Cox) test was performed to evaluate significance of survival curves and to calculate the hazard ratio (HR=10.64) of $OPN^{ko/ko}$ chimeras. ** $p<0.01$. **(F)** Representative colonoscopy images, **(G)** longitudinal disease activity index, and **(H)** Kaplan-Meier survival curves of colitis-susceptible IL-$10^{ko/ko}$ mice treated with vehicle, MMP14i NSC405020 (3) (2.0 mg/kg/day, 3 times per week), or anti-OPN neutralizing mAb (clone 103D6 (8), 5.0 mg/kg/day, daily) showed significant adverse effect of OPN neutralization leading to severe colitis, failure to resolve inflammation, and death following acute colitis and resolution period. One-way ANOVA with Dunnett's multiple comparison test was performed to compare difference in disease index of MMP14i or anti-OPN treatments with vehicle group (n=5-8 mice per condition, * $p<0.05$, ** $p<0.01$, *** $p<0.001$, ns non-significant). A log-rank (Mantel-Cox) test was performed to assess survival differences and calculate the hazard ratio (HR=31.11) of OPN neutralization in IL-$10^{ko/ko}$ mice (*** $p<0.001$). **(I)** Quantification of percent weight loss, **(J)** colon length, and **(K)** spleen weight of individual IL-$10^{ko/ko}$ versus wildtype mouse

at survival endpoints indicating heightened disease severity of OPN neutralization (one-way ANOVA with Tukey's multiple comparison test). **(L)** Quantification of percent weight loss, **(M)** colon length, **(N)** colon weight, **(O)** spleen weight, **(P)** BM counts, and **(Q)** PMN counts of PDX-bearing NGS mice (TM00170) following 28-day treatment with vehicle (PEG300, isotype control) or MMP14i (2.0 mg/kg/day, 3 times per week) (one-way ANOVA with Tukey's multiple comparison test, n=4-6 mice, ns non-significant). Data are presented as mean ± SEM.

Figure S10

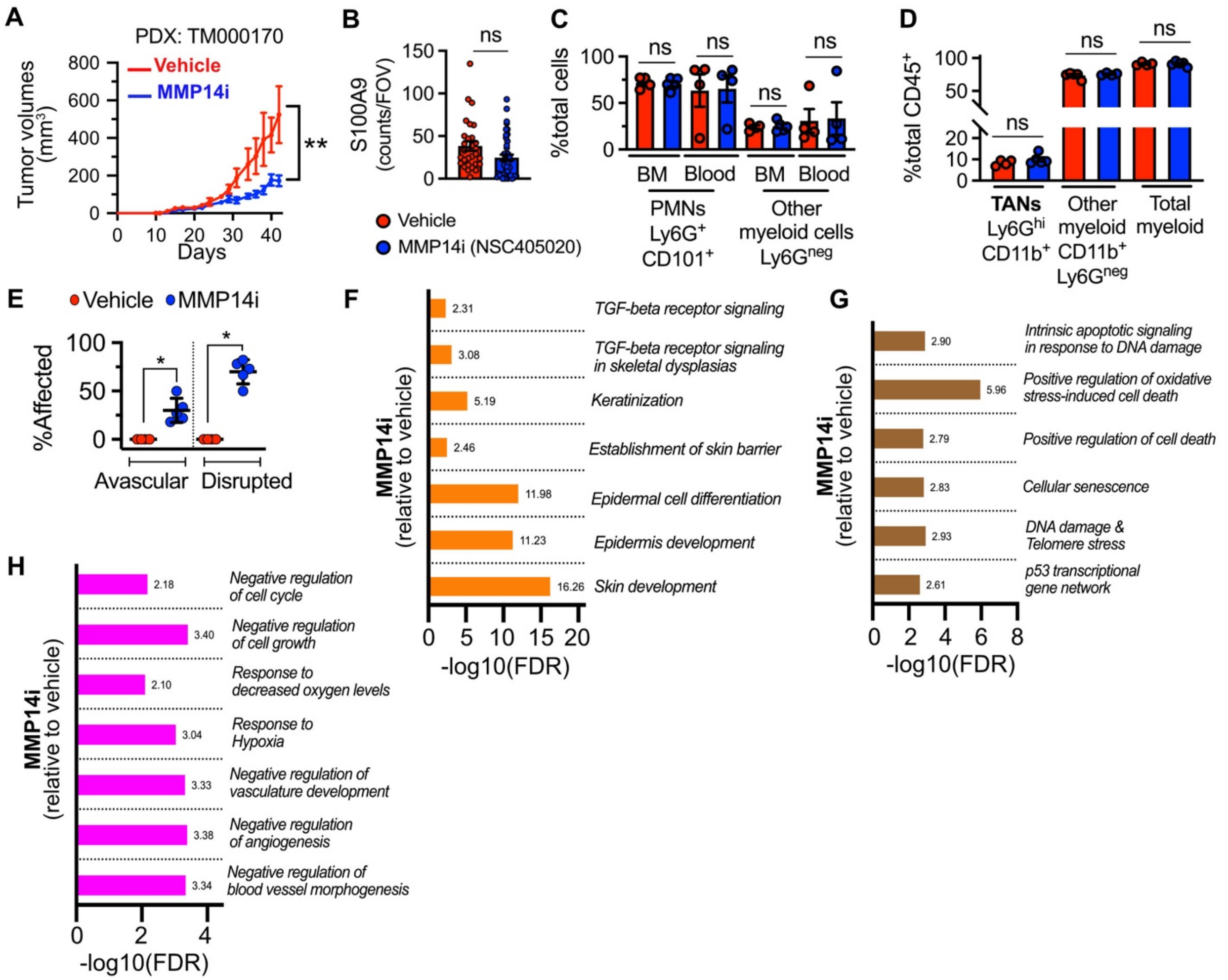

Supplemental Figure 10: Defining phenotypic and transcriptional changes in PDX tumors following MMP14 inhibition. (A) Tumor curves presented as mean ± SD of the spaghetti plots shown in Figure 7B. **(B)** Quantification of S100A9-positive cells in all analyzed PDX tumors (n=30-50 FOVs for each analyzed tumor per condition, two-tailed unpaired student's t test, ns non-significant). **(C)** Percentage of PMNs (Ly6G$^+$/CD11b$^+$) and other myeloid cells in BM and peripheral blood of PDX-bearing mice with specified treatments. **(D)** Percentage of TANs, other myeloid, and total myeloid cells in PDX tumors (vehicle: n=4, MMP14: n=5, Mann-Whitney's U test, * p<0.05, ns non-significant). **(E)**

Percentage of tumor FOVs with avascular phenotypes and disrupted vessel morphologies with each specified treatment (Mann-Whitney's U test, ** $p<0.01$, *** $p<0.001$, **** $p<0.0001$). Data are presented as mean ± SD. **(F)** TGF-beta signaling and keratinization, **(G)** cell stress and apoptosis, **(H)** negative regulations of cell cycle and cell growth and vasculature development.

Figure S11

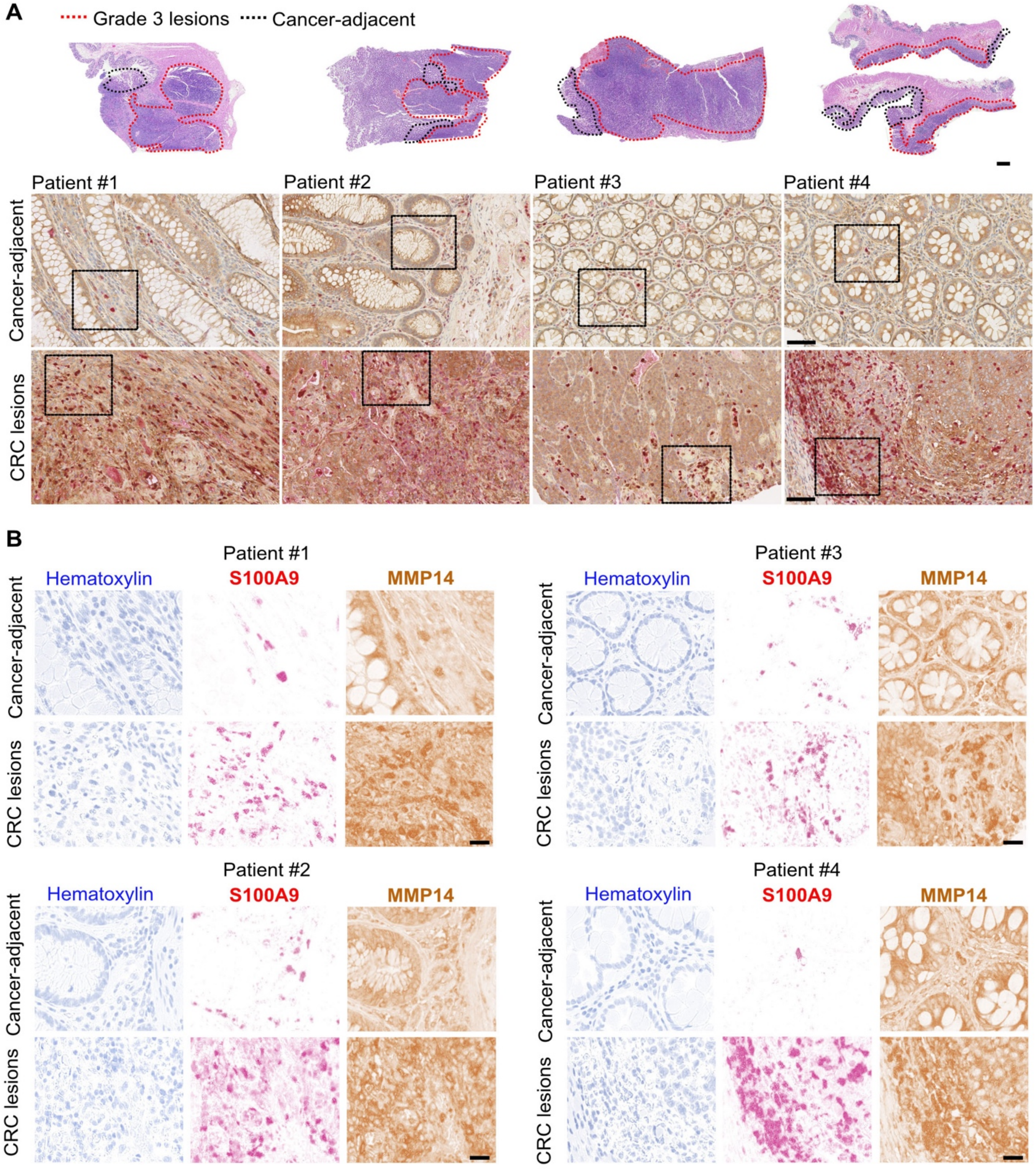

Supplemental Figure 11: Identification of MMP14-positive TANs in high-grade CRC patients. (A) Top panel shows representative H&E images of four high-grade CRC patients (grade 3, stage III-IV). Scale bar 500μm. Outlines specify hyperchromatic regions

of cancer lesions (red) and cancer adjacent regions of healthy, non-dysplastic crypt epithelia (black). Matched slides were sequentially stained for S100A9 (red) and MMP14 (brown) by IHC. Middle panel shows cancer-adjacent regions with mostly MMP14-negative S100A9-positive PMN cells. Bottom panel shows intratumor CRC regions with strong MMP14 staining and accumulation of MMP14-positive S100A9-positive TANs. Scale bar 100μm. Insets identify regions of interest for magnification and three-color deconvolution. **(B)** Digital color deconvolution of IHC images (insets shown in A) using the three-color Fast Red-Fast Blue-DAB Vector (red, brown, blue). Deconvolution into three color channels identify strong dual staining of S100A9 and MMP14 within CRC lesions of all four CRC patients. Scale bars 25μm.

References

1. Gorio A, et al. Reparixin, an inhibitor of CXCR2 function, attenuates inflammatory responses and promotes recovery of function after traumatic lesion to the spinal cord. *J Pharmacol Exp Ther*. 2007;322(3):973–981.

2. Kim HY, et al. Reparixin, an inhibitor of CXCR1 and CXCR2 receptor activation, attenuates blood pressure and hypertension-related mediators expression in spontaneously hypertensive rats. *Biol Pharm Bull*. 2011;34(1):120–127.

3. Remacle AG, et al. Novel MT1-MMP small-molecule inhibitors based on insights into hemopexin domain function in tumor growth. *Cancer Res*. 2012;72(9):2339–2349.

4. De Robertis M, et al. The AOM/DSS murine model for the study of colon carcinogenesis: From pathways to diagnosis and therapy studies. *J Carcinog*. 2011;10:9.

5. Bui TM, et al. Neutrophils Alter DNA Repair Landscape to Impact Survival and Shape Distinct Therapeutic Phenotypes of Colorectal Cancer. *Gastroenterology*. [published online ahead of print: March 19, 2021]. https://doi.org/10.1053/j.gastro.2021.03.027.

6. Boivin G, et al. Durable and controlled depletion of neutrophils in mice. *Nat Commun*. 2020;11(1):2762.

7. Arulanandam R, et al. VEGF-Mediated Induction of PRD1-BF1/Blimp1 Expression Sensitizes Tumor Vasculature to Oncolytic Virus Infection. *Cancer Cell*. 2015;28(2):210–224.

8. Klement JD, et al. Osteopontin Blockade Immunotherapy Increases Cytotoxic T Lymphocyte Lytic Activity and Suppresses Colon Tumor Progression. *Cancers (Basel)*. 2021;13(5):1006.

9. Bordeleau F, et al. Matrix stiffening promotes a tumor vasculature phenotype. *Proc Natl Acad Sci U S A*. 2017;114(3):492–497.

10. Rehring JF, et al. Released Myeloperoxidase Attenuates Neutrophil Migration and Accumulation in Inflamed Tissue. *Front Immunol*. 2021;12:654259.

11. Batra A, et al. Experimental Colitis Enhances Temporal Variations in CX3CR1 Cell Colonization of the Gut and Brain Following Irradiation. *Am J Pathol*. 2022;192(2):295–307.

12. Martin M. Cutadapt removes adapter sequences from high-throughput sequencing reads. *EMBnet j*. 2011;17(1):10.

13. Dobin A, et al. STAR: ultrafast universal RNA-seq aligner. *Bioinformatics*. 2013;29(1):15–21.

14. Anders S, Pyl PT, Huber W. HTSeq--a Python framework to work with high-throughput sequencing data. *Bioinformatics*. 2015;31(2):166–169.

15. Love MI, Huber W, Anders S. Moderated estimation of fold change and dispersion for RNA-seq data with DESeq2. *Genome Biol*. 2014;15(12):550.

16. Ritchie ME, et al. limma powers differential expression analyses for RNA-sequencing and microarray studies. *Nucleic Acids Res*. 2015;43(7):e47.

17. Wang X, et al. HTSanalyzeR: an R/Bioconductor package for integrated network analysis of high-throughput screens. *Bioinformatics*. 2011;27(6):879–880.

18. Zhou Y, et al. Metascape provides a biologist-oriented resource for the analysis of systems-level datasets. *Nat Commun*. 2019;10(1):1523.

19. Veglia F, et al. Analysis of classical neutrophils and polymorphonuclear myeloid-derived suppressor cells in cancer patients and tumor-bearing mice. *J Exp Med*. 2021;218(4):e20201803.

20. Xie X, et al. Single-cell transcriptome profiling reveals neutrophil heterogeneity in homeostasis and infection. *Nat Immunol*. 2020;21(9):1119–1133.

21. Khoyratty TE, et al. Distinct transcription factor networks control neutrophil-driven inflammation. *Nat Immunol*. 2021;22(9):1093–1106.

22. Goveia J, et al. An Integrated Gene Expression Landscape Profiling Approach to Identify Lung Tumor Endothelial Cell Heterogeneity and Angiogenic Candidates. *Cancer Cell*. 2020;37(1):21-36.e13.

23. Carpentier G, et al. Angiogenesis Analyzer for ImageJ - A comparative morphometric analysis of “Endothelial Tube Formation Assay” and “Fibrin Bead Assay.” *Sci Rep*. 2020;10(1):11568.

24. Wiesolek HL, et al. Intercellular Adhesion Molecule 1 Functions as an Efferocytosis Receptor in Inflammatory Macrophages. *Am J Pathol*. 2020;190(4):874–885.

25. Butin-Israeli V, et al. Neutrophil-induced genomic instability impedes resolution of inflammation and wound healing. *J Clin Invest*. 2019;129(2):712–726.

26. Gao J, et al. Integrative analysis of complex cancer genomics and clinical profiles using the cBioPortal. *Sci Signal*. 2013;6(269):pl1.

27. Evrard M, et al. Developmental Analysis of Bone Marrow Neutrophils Reveals Populations Specialized in Expansion, Trafficking, and Effector Functions. *Immunity*. 2018;48(2):364-379.e8.
